# Neovascularization of engineered tissues for clinical translation: Where we are, where we should be?

**DOI:** 10.1063/5.0044027

**Published:** 2021-04-05

**Authors:** Muhammad Anwaar Nazeer, Ismail Can Karaoglu, Onur Ozer, Cem Albayrak, Seda Kizilel

**Affiliations:** 1 Chemical and Biological Engineering, Koç University, Istanbul 34450, Turkey; 2 Reserach Center for Translational Medicine, Koç University, Istanbul 34450, Turkey; 3 Biomedical Sciences and Engineering, Koç University, Istanbul 34450, Turkey; 4 Department of Biotechnology, Beykoz Institute of Life Sciences and Biotechnology (BILSAB), Bezmialem Vakıf University, Istanbul 34820, Turkey

## Abstract

One of the key challenges in engineering three-dimensional tissue constructs is the development of a mature microvascular network capable of supplying sufficient oxygen and nutrients to the tissue. Recent angiogenic therapeutic strategies have focused on vascularization of the constructed tissue, and its integration *in vitro*; these strategies typically combine regenerative cells, growth factors (GFs) with custom-designed biomaterials. However, the field needs to progress in the clinical translation of tissue engineering strategies. The article first presents a detailed description of the steps in neovascularization and the roles of extracellular matrix elements such as GFs in angiogenesis. It then delves into decellularization, cell, and GF-based strategies employed thus far for therapeutic angiogenesis, with a particularly detailed examination of different methods by which GFs are delivered in biomaterial scaffolds. Finally, interdisciplinary approaches involving advancement in biomaterials science and current state of technological development in fabrication techniques are critically evaluated, and a list of remaining challenges is presented that need to be solved for successful translation to the clinics.

## I. INTRODUCTION

Globally, cardiovascular disease is one of the major causes of mortality, accounting for approximately 30% of adult fatalities in developed countries.^1^ Numerous pathological conditions, such as various types of cancer, macular degeneration, and rheumatoid arthritis, are related to cardiovascular and angiogenic diseases.^2^ Inadequate blood supply and disrupted blood vessels often lead to peripheral arterial and myocardial ischemia conditions, cerebrovascular, and coronary artery disease.^1^ Tissue engineering aims to reconstruct tissues and organs as artificial replacements, thereby addressing expensive and prevalent health problems. Regenerative medicine aims to circumvent issues associated with organ transplants such as graft-vs-host disease (GvHD) and organ shortage.^3^ Several avascular tissues, such as cartilage, bladder, and skin, have already been constructed successfully and been used in clinics.^4–6^ Unfortunately, tissue engineering strategies for larger vascularized organs and thick tissues have thus far proven limited, due to the lack of standardized protocols for generating a robust microvascular network with a mean diffusion distance of 150–200 *μ*m, a critical diffusion limit. This diffusion range is critical for sufficient nutrient and gas exchange in more complex tissues and organs such as liver, heart, muscle, and bone.^7^ Therapeutic angiogenesis aims to address this issue by enhancing the formation of new blood vessels (neovascularization) in engineered tissues.

**FIG. 1.**
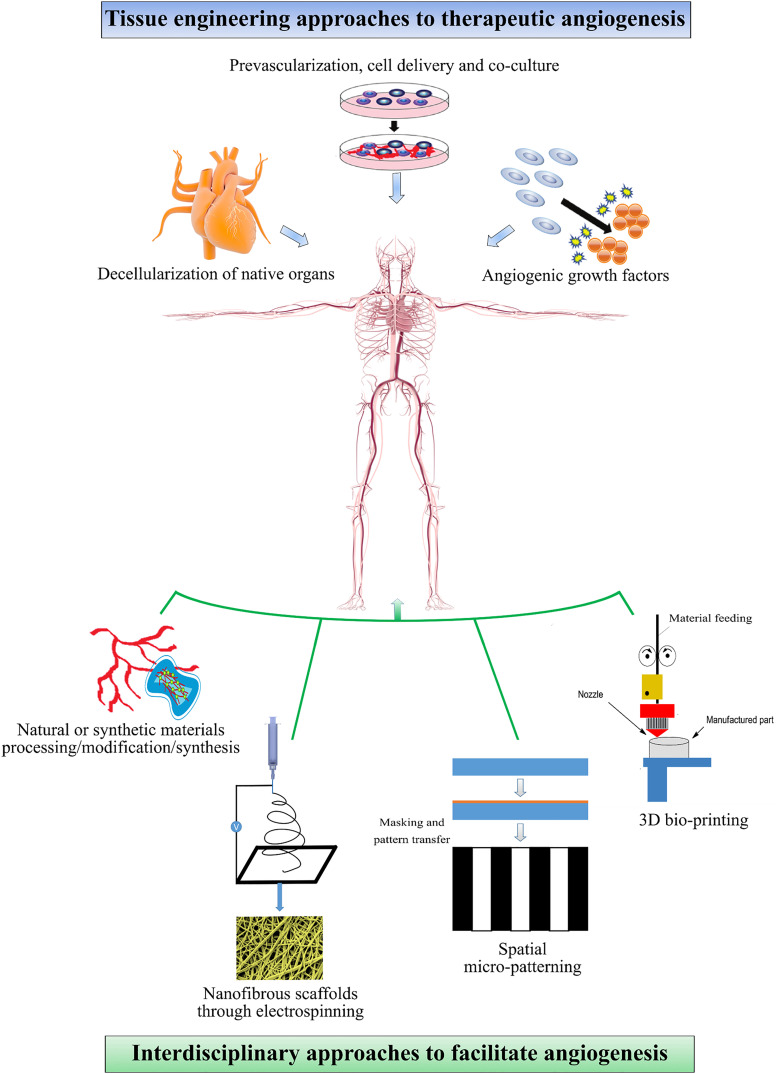
Schematic representation of tissue engineering strategies for therapeutic angiogenesis and interdisciplinary approaches to facilitate angiogenesis. Decellularization of native organs has been used to obtain immuno-compatible tissues as a scaffold where a suitable ECM matrix is present. Neovascularization can be induced by using pre-vascularization, *in vivo* cell delivery, and co-culturing strategies or delivering angiogenic growth factors. Different techniques such as electrospinning, spatial micropatterning, 3D printing have been used to facilitate angiogenesis at the desired site.

Molecular interactions between growth factors (GFs), regenerative cells, natural extracellular matrix (ECM) components, and biomaterial scaffolds have been investigated to replace failed tissues and enhance neovascularization at targeted sites for therapeutic purposes. Considering the capacity of this natural microenvironment to dynamically regulate angiogenesis, mimicking the 3D natural microenvironment of tissues has become a key aspect of regenerative medicine. In the literature, the topic of vascularization of engineered tissues has been discussed by several groups in the scope of either delivery of GFs or manufacturing micro/macrovessel structures that facilitate the local vascularization.^8–12^ In this review, we cover promising and recent therapeutic angiogenesis strategies, which feature various combinations of the following subjects: (a) delivery of vascular [e.g., vascular endothelial cells (ECs)] and regenerative cell types (e.g., vascular progenitor cells, stem cells, etc.), (b) local and sustained delivery of angiogenic GFs, and (c) modified natural or synthetic biomaterial scaffolds that mimic the natural microenvironment and localize angiogenic GFs to the target site. Combination of important parameters approach is required to utilize tissue engineering techniques (cells, decellularized tissue, and GFs) and inter-disciplinary systems (functionalized-biomaterials and fabrication techniques) together to develop a successful system for clinical translation of engineered tissues/organs (Fig. 1).

### A. Microvasculature and neovascularization

**FIG. 2.**
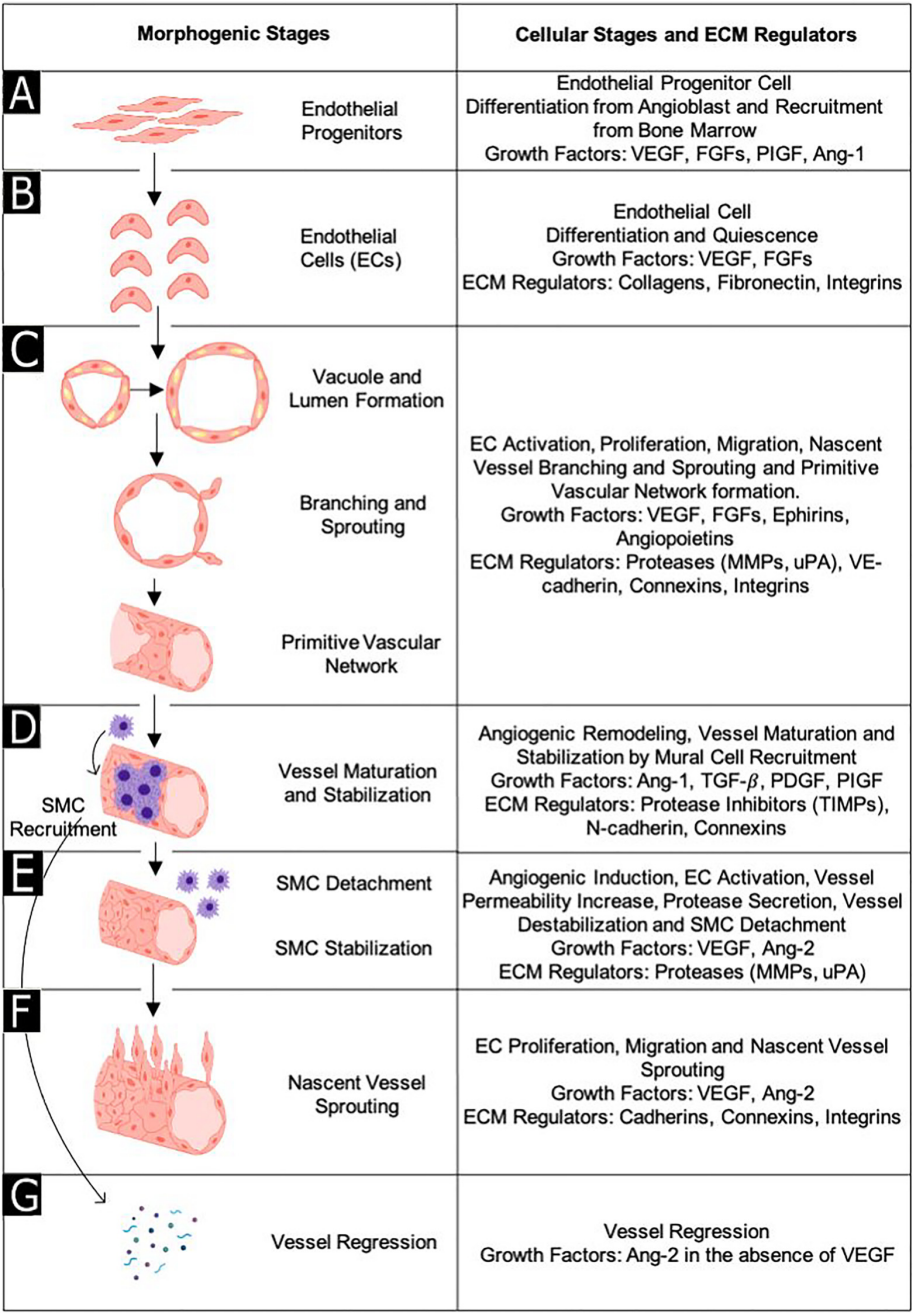
Morphogenetic stages with major angiogenic regulatory growth factors and extracellular matrix molecules involved in neovascularization. Endothelial progenitors are differentiated from mesodermal cells as first step of vasculogenesis (a). EPCs differentiates into ECs (b). Organization of primitive vascular network are achieved by vacuole and lumen formation, branching and sprouting (c). Vessel is matured and stabilized by recruitment of SMC (d). Vessel destabilization occurs owing to SMC detachment (e). Nascent vessel sprouting occurs with the migration of endothelial cells up a growth factor gradient in response to biochemical growth factors (f). Vessel regression is observed during tissue repair and regeneration (g). Reprinted by permission from M. P. Lutolf and J. A. Hubbell, Nat. Biotechnol. **23**(1), 47–55 (2005). Copyright 2005 Springer Nature Customer Service Center GmbH, Springer Nature.

While conducting vascularization strategies with 3D tissue constructs, the main aim is to mimic the *in vivo* microvascular architecture and angiogenic processes for a proper therapeutic vascularization. Therefore, the top prerequisite is to gain a better understanding of microvascular biology along with its key events and components, such as the role of the ECM molecules and GFs. Microvasculature is the system of small blood vessels (microvessels) within a tissue, and it consists of the endothelium, the basement membrane, and supporting mural cells. The endothelium is the epithelial layer composed of ECs that lines the inner surface of blood vessels (i.e., arteries, veins, and capillaries).^13^ It is thrombo-resistant, and it functions as a semi-permeable barrier to control blood circulation.^14^ The basement membrane comprises the ECM and contributes to angiogenesis by harboring membrane proteins that promote differentiation of ECs and the development of blood vessels.^15^ Finally, the surrounding mural cells, i.e., pericytes and smooth muscle cells (SMCs), stabilize, and mature new vessels.^16^ Neovascularization refers to the formation and growth of new blood vessels through several vascular processes, namely vessel formation, sprouting, maturation, stabilization, remodeling, and specialization (Fig. 2).^17^ It includes both vasculogenesis and angiogenesis. Vasculogenesis describes the de novo formation of blood vessels and the establishment of primitive vasculature during embryogenesis that includes differentiation of EC precursors and their subsequent assembly into a vascular network.^16^ In the first step of vasculogenesis [Fig. 2(a)], mesodermal cells differentiate into EC precursors, namely, angioblasts and hemangioblasts.^18^ Then, these precursors give rise to endothelial progenitor cells (EPCs).^19^ EPCs originate in the bone marrow and are released into peripheral blood to differentiate into mature ECs^20^ [Fig. 2(b)]. These mature ECs proliferate and are subsequently organized into a primitive vascular network^21^ [Fig. 2(c)]. In the last stage of vasculogenesis, primitive vasculature undergoes angiogenic remodeling, which is the transformation of this primary vasculature by vessel enlargement and pruning into an interconnected branching network.^21^ Finally, the remodeled vasculature is matured and stabilized by the recruitment of mural cells (pericytes and SMCs) and surrounding ECM molecules (e.g., integrins, cadherins, connexins)^21^ [Fig. 2(d)]. Angiogenesis is the formation and growth of new blood vessels through sprouting from existing microvasculature during embryogenesis, postnatal life as well as in disease pathology. In early angiogenesis, physiological and pathological stimuli such as inflammation, hypoxia, and ischemia induce the expression of angiogenic GFs and nitric oxide synthase (NOS) through the activation of hypoxia-inducible factor 1α (HIF-1α).^22^ NOS produces nitric oxide (NO), causing vasodilation of existing vessels, while soluble angiogenic GFs form a gradient in the stimulus zone that activates ECs and increases vessel permeability. Increased vessel permeability forms a provisional scaffold. As initial destabilization of mature vessels is required for sprouting, activated ECs (tip ECs) secrete various proteases to degrade the basement membrane and ECM. Vessel destabilization is completed by SMC detachment [Fig. 2(e)].

ECs then migrate into the zone of the GF gradient and lead to the sprouting of nascent blood vessels from existing ones.^18,20^ Subsequently, ECs form vacuoles via phagocytosis and pinocytosis, and nascent vacuoles fuze to form a lumen in long vessel extensions^23^ [Fig. 2(f)]. If critical GFs such as vascular endothelial growth factor (VEGF), platelet-derived growth factor (PDGF), or transforming growth factor (TGF) are not present in the microenvironment, vessel regression occurs instead of sprouting [Fig. 2(g)]. Since the new vasculature that originates from existing blood vessels is immature; nascent blood vessels must undergo subsequent maturation, stabilization, and remodeling, similar to the last stage of vasculogenesis [Fig. 2(d)]. The new vasculature is stabilized and matured by the inhibition of EC proliferation, the establishment of the new basement membrane, and encapsulation by recruited supportive mural cells (i.e., pericytes and SMCs). Pericytes and SMCs stabilize and mature new vessels through inhibition of EC proliferation and migration, protection against vessel regression and rupture, ECM production, as well as the relaxation and contraction of blood vessels for the control of permeability and circulation^22^ [Fig. 2(g)]. Finally, ECs become quiescent again with long-term survival capacity. In the end, an intact branched vascular network with a maximum distance of 150–200 *μ*m between capillaries emerges for proper vascular activity.^7^

## II. TISSUE ENGINEERING STRATEGIES FOR THERAPEUTIC ANGIOGENESIS

In therapeutic angiogenesis, the main aim is to recapitulate the natural microenvironment and the pertinent molecular processes in engineered tissue constructs to promote neovascularization and create new vessel ingrowth in ischemic tissues. Three main approaches are being used to promote vascularization/angiogenesis in tissue engineering, as are explained in detail below: (1) decellularization, (2) cell-based strategies focused on vascular and regenerative cells, (3) angiogenic GFs addition.

### A. Decellularization

Isolation of tissues/cells from cadaveric human donors is a relatively fast and straightforward procedure to obtain immuno-compatible tissues. Large-volume tissues/organs can also be obtained from animals. Antigenic cellular constituents can be removed by sequential perfusion decellularization technique while retaining the natural ECM components. The basic principle involves; exposure of harvested tissue or organ to a dilute surfactant solution, rinsing-off cellular components followed by recellularization.^24,25^ The advantage of this technique is that it provides the native architecture of ECM and can eliminate the need for vascularization through cell implantation.^26^ The angiogenic efficiency of decellularized tissues/organs can also be augmented by the co-culture of cells to facilitate vascularization. For example, Dew *et al.* used decellularized rat intestine as an *in vitro* model to investigate neovascularization potential. During decellularization, almost 90% of cellular components were removed while retaining vascular channels. Recellularization was performed by using human dermal microvascular endothelial and human dermal fibroblast cells. In the presence of pro-angiogenic GF like VEGF, neovascularization, and the sprouting effect was observed.^27^

Even though the decellularization technique provides a suitable ECM matrix, it is still associated with some constraints such as the antigenicity from xenogeneic tissues and dependence on donor tissues, which are not readily available. However, these matrices can be used as a template for a better understanding of native ECM structure, and alternatives should be explored for developing an engineered tissue/organ possessing vascularized architecture.

### B. Cell-based strategies

The main objective in cell-based strategies is to induce neovascularization by transplanting cells into the targeted site using either one or a combination of the following methods: (1) pre-vascularization, which requires vascularization of the tissue construct before transplantation, (2) *in vivo* cell transplantation that involves vascularization within the microenvironment of the implanted tissue, and (3) design of engineered heterocellular organoids to retrieve tissue function.

#### 1. Pre-vascularization

**FIG. 3.**
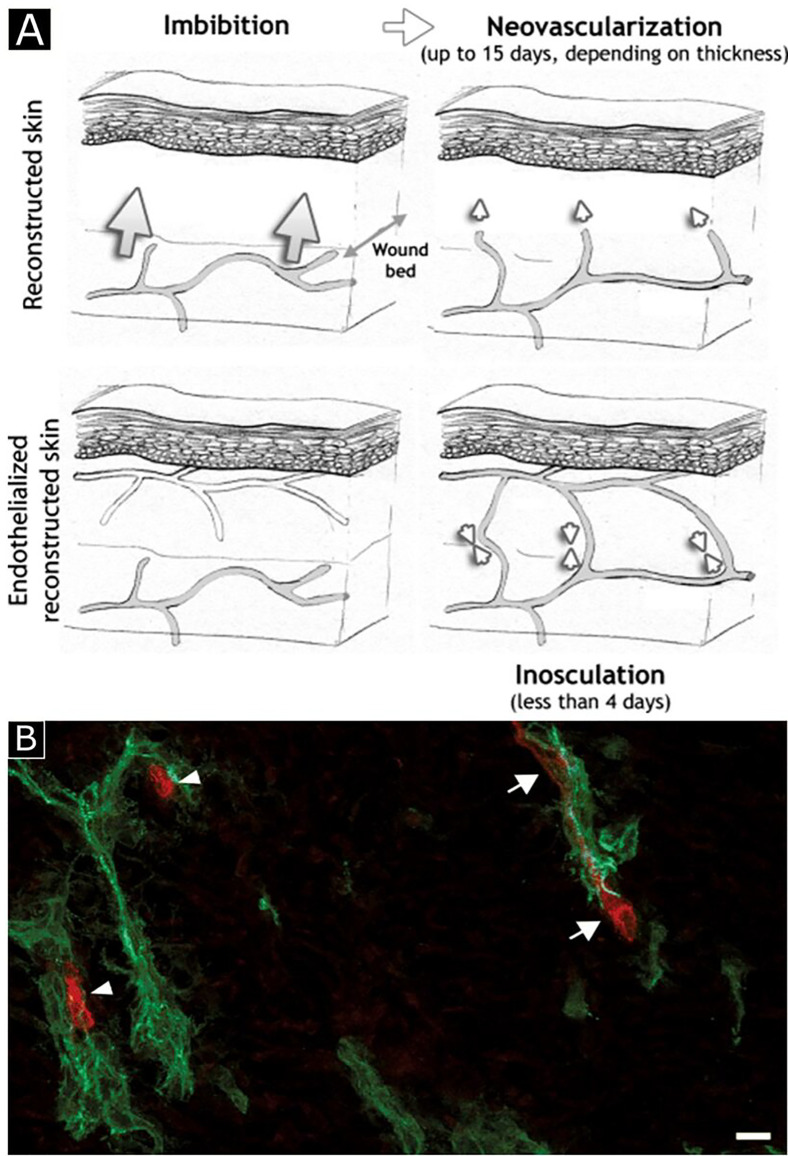
Schematic illustration of three different processes involved in graft nutrition (a). *In vitro* revascularization (b). Endothelialized capillary-like tubes are formed in a skin construct. Human (red) and mouse ECs (green) were co-localized (arrows) or branched (arrowheads). Reprinted with permission from Tremblay *et al.*, Am. J. Transplant. **5**, 1002 (2005). Copyright 2005 John Wiley & Sons, Inc.

Pre-vascularization can be utilized in both *in vivo* and *in vitro* approaches. In *in vitro* pre-vascularization, ECs are seeded into scaffolds and cultured to generate a three-dimensional tissue construct that includes a branched vessel network. This primitive vasculature construct is then transplanted into the ischemic region to create a connection with existing host microvasculature, in a process called anastomosis.^28^ Neovascularization is a delayed process (almost 15 days) and depends on graft thickness. Whereas inosculation, the formation of functional connections with host capillaries,^29^ is a thickness independent and fast process (<4 days) [Fig. 3(a)]. Therefore, the advantage of *in vitro* pre-vascularization is the possibility to overcome the limitations associated with delayed vascularization, such as being able to develop a vascular network much faster than that during natural healing of a burned, damaged, or thick ischemic tissue. For example, pre-vascularization of collagen tissue was achieved through *in vitro* seeding of ECs, fibroblasts, and keratinocytes to accelerate the vascularization process. The researchers observed the formation of endothelialized capillary-like tubes and a well-established microvessel network in *in vitro*. Upon implantation of the resulting pre-vascularized construct into nude mice, the connection with host tissue was rapidly achieved within four days compared to non-endothelialized control, which required 14 days for anastomosis [Fig. 3(b)]. The host blood vessel's ingrowth results in vascularization of the whole graft, and the duration depends on the graft thickness.^30^ During *in vivo* pre-vascularization, the engineered acellular tissue construct is implanted into the host to promote vascularization, which differs it from *in vitro* pre-vascularization where pre-vascularization is performed before implantation. After the first transplantation, host cells (e.g., ECs and fibroblasts) infiltrate into the tissue construct and form perfusable microvessels within the implant. Then, secondary surgery is conducted to retrieve the vascularized construct from the host, and the construct is subsequently implanted into the diseased site of the same host.^7^

**FIG. 4.**
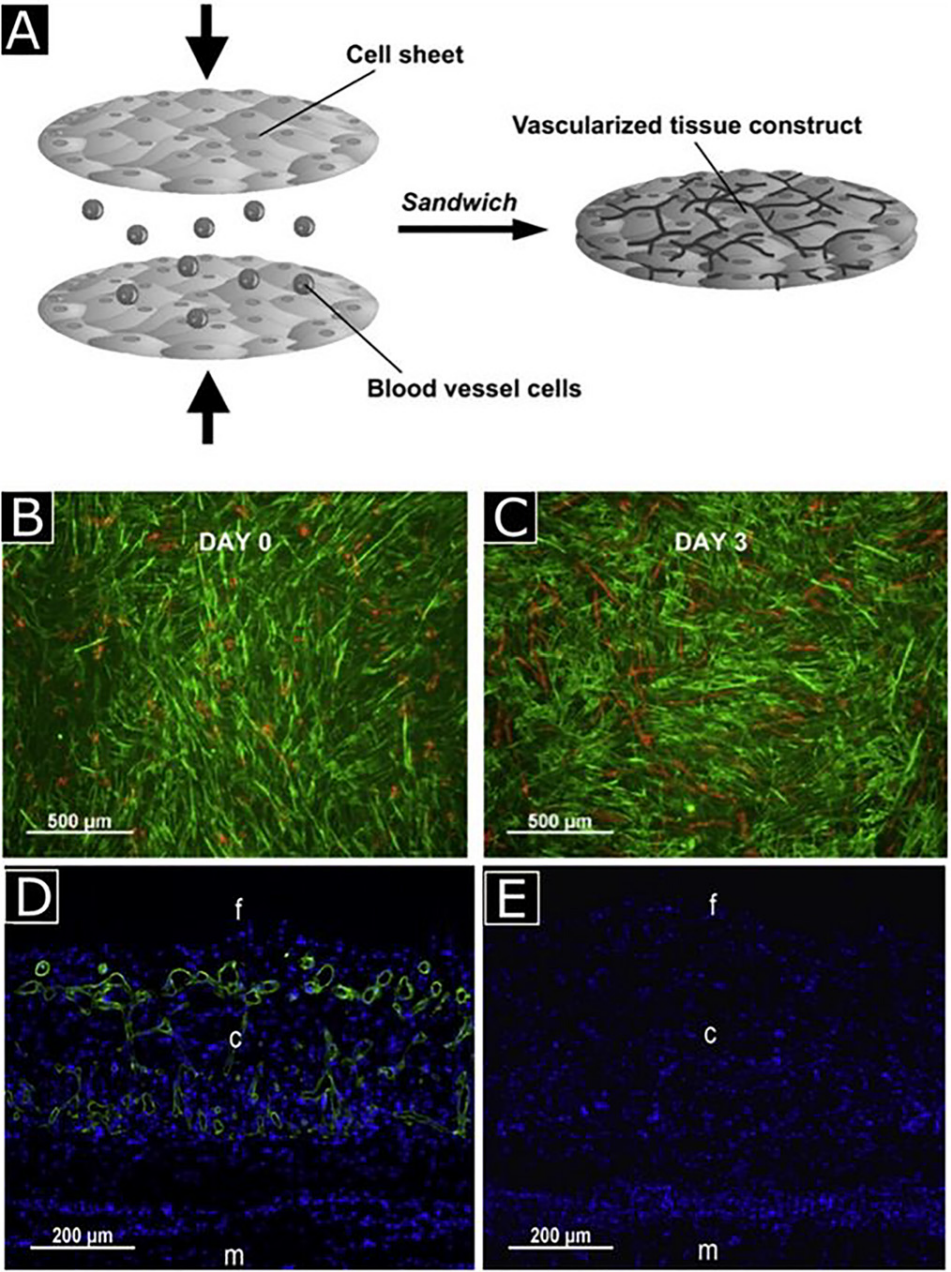
Schematic representation for vascularized tissue formation by sandwich method (a) human umbilical vein endothelial cells (HUVECs) sandwiched between myoblast sheets with the help of a gelatin-coated plunger, cultured up to 3 days, and stained with UEA-I (red) and anti-desmin antibody (green) for HUVECs and myoblasts, respectively (b) and (c). Observation of neovascularization with anti-human CD31 antibody (green) staining in five-layered myoblast sheet constructs with (d) and without HUVECs (e). Notations f, c, and m represent the fibrin gel, cell sheet construct, and muscle tissue, respectively. Reprinted with permission from Sasagawa *et al.*, Biomaterials **31**, 1646 (2010). Copyright 2010 Elsevier.

There are different ways to induce pre-vascularization, and one of the commonly used methods is called cell-sheet technology in which stacks of cell monolayers (e.g., ECs, cardiomyocytes, SMCs) are implanted in the form of a sheet into the ischemic region. This implantation promotes neovascularization along with high blood perfusion *in vivo.*^7,31^ In one implementation, the EC network was sandwiched between myoblast sheet constructs with the help of a gelatin-coated plunger [Fig. 4(a)]. The developed sandwiched construct was then cultured in a tissue culture plate [Figs. 4(b) and 4(c)]. This pre-vascularized tissue construct led to a functional connection with host microvasculature *in vivo*. In addition, implantation of cardiomyocyte sheets with EC monolayers promoted neovascularization in myocardial infarction and accelerated cardiac function repair *in vivo* by creating a highly vascularized 3D cardiac tissue network [Figs. 4(d) and 4(e)].^32^

Finally, arteriovenous (AV) shunt loop can be used for *in vivo* pre-vascularization, where a shunt loop is formed between artery and vein in the AV loop chamber that is either empty or contains an ECM scaffold. Then, the chamber is inserted into a region rich in vessels to generate vascular network construct *in vivo*. The resulting construct is transplanted into the ischemic region to induce angiogenesis by the increased shear stress and tension of the wall within the loop's vasculature.^7,33,34^ Both *in vitro* and *in vivo* pre-vascularization techniques are successful in providing functional connections between the pre-vascularized tissue construct and the host circulatory system, where host microvessel ingrowth into the tissue construct is not necessary. In addition, *in vivo,* pre-vascularization requires multiple surgeries, while blood perfusion and rate of neoangiogenesis are too slow due to the absence of microsurgical connections. The latter leads to insufficient oxygen and nutrient supply for the maintenance of vessels within the scaffold, which could ultimately lead to disruption of blood flow and vessel regression.^35^

#### 2. Regenerative cell delivery and co-culture of cells

Another cell-based strategy for inducing neovascularization comprises the delivery of regenerative cells alone or in combination with supporting cells into the ischemic zone. Generally, ECs, EPCs, hematopoietic stem cells (HSCs), and mesenchymal stem cells (MSCs) are used to promote angiogenesis. Their combination can mimic embryonic neovasculogenesis, where, a pericyte (PC)-stabilized capillary bed network is assembled by angioblasts, EPCs, ECs, and via mural cell formation by MSCs.^36^

**FIG. 5.**
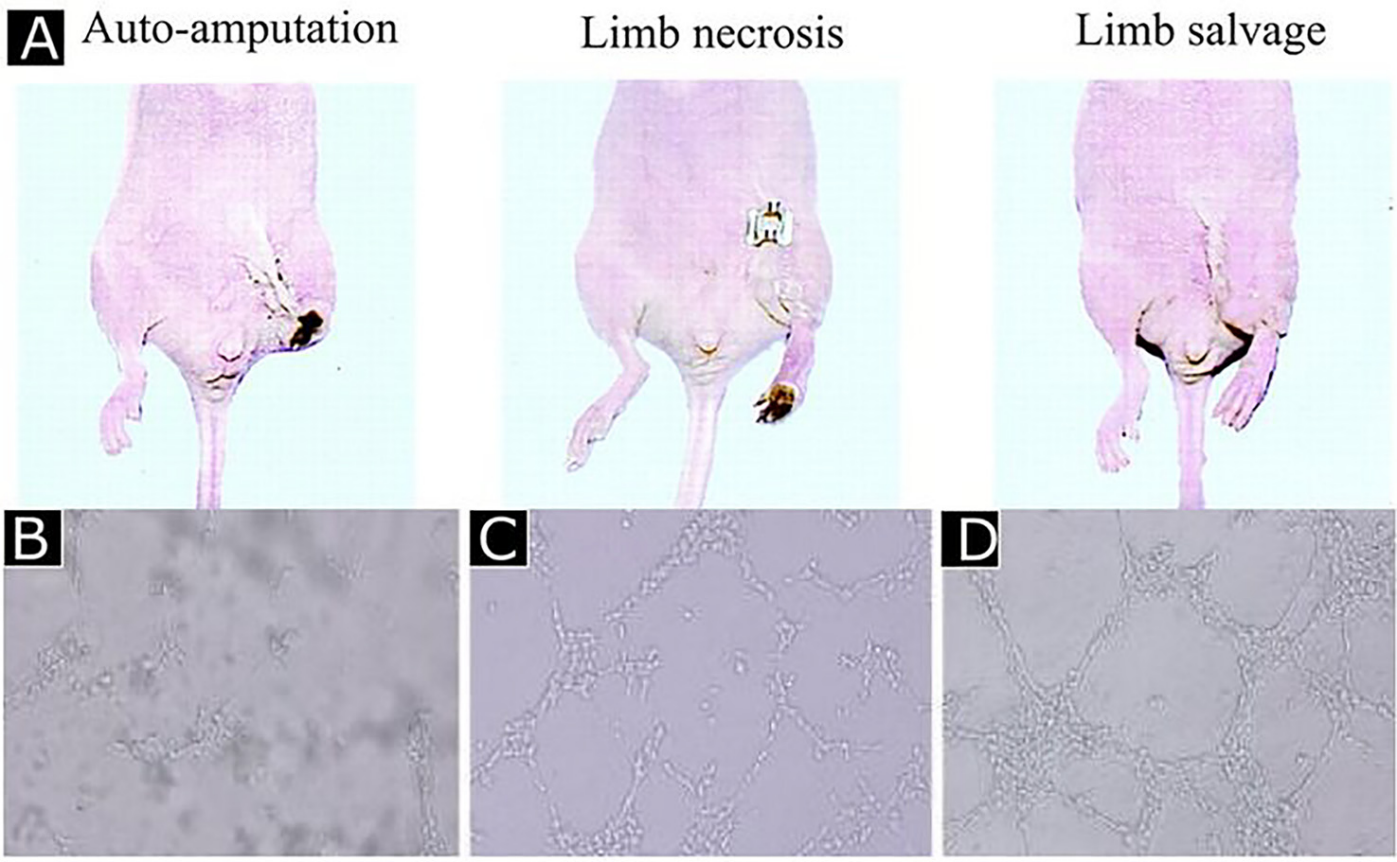
Human endothelial progenitor cells improved limb salvage. (a) Reprinted with permission from Kalka *et al.*, Proc. Natl. Acad. Sci. U. S. A. **97**, 3422 (2000). Copyright 2000 National Academy of Sciences, USA. The co-culture of HUVECs and MSCs led to high tubulogenesis (c) and (d) compared to HUVEC only culture (B). Reprinted by permission from Zhang *et al.*, J. Huazhong Univ. Sci. Technol., Med. Sci. **32**, 173–180 (2012). Copyright 2012 Springer Nature Customer Service Center GmbH, Springer Nature.

Co-culture systems can promote neovasculogenesis and vessel organization because of the interactions between ECs and other cell types. In the treatment of avascular necrosis of the femoral head, co-culture of human MSCs (hMSCs) and HUVECs was shown to mimic the microvascular microenvironment successfully; augment HUVEC migration, survival, and angiogenesis; and inhibit apoptosis after hMSCs transplantation [Fig. 5(b)].^37^ Autologous stem cells can also be used to form engineered vascular constructs. Bone marrow-derived EPCs can circulate and incorporate themselves into vessel walls. EPCs have been shown to give rise to ECs, SMCs, pericytes, and they can contribute to microvasculature development when injected into chick embryos.^38^ Implantation of human EPCs into nude mice with hindlimb and myocardial ischemia, successfully restored blood flow, reduced limb loss, enhanced capillary density [Fig. 5(a)],^39^ reduced myocardial fibrosis and protected left ventricle function.^40^ The lack of network guidance through biochemical and biophysical cues limits co-culture systems that are required to achieve an organized microvascular architecture. These limitations prevent the formation of ordered vascular networks.^41^ Moreover, since neovascularization is also orchestrated by the vascular microenvironment, ECM-bound, and soluble signals in addition to the cells, the delivery of regenerative cells alone was insufficient to obtain a viable vascular network. This approach was more recently improved by the introduction of GFs into biomaterial scaffolds to achieve potent angiogenic therapy.

#### 3. Design of engineered heterocellular organoids to retrieve tissue function

Organoids are 3D cellular organizations that are commonly used as an intermediate step between conventional tissue culture and animal experiments. Until recently, organoids have been prepared with a single cell type, which does not mimic the real microenvironment of the native tissue. Heterocellular organoids have been prepared with different cell types such as stem cells or immune cells to investigate the effects of cells on the organoid function or graft acceptance.^42–44^ Here, we will not be addressing all types of cells that can be used in generating heterocellular organoids. A piece of quite wide and explanatory information can be found in our recent study, particularly for the design of implantable insulin-secreting heterocellular islet organoids.^45^

**FIG. 6.**
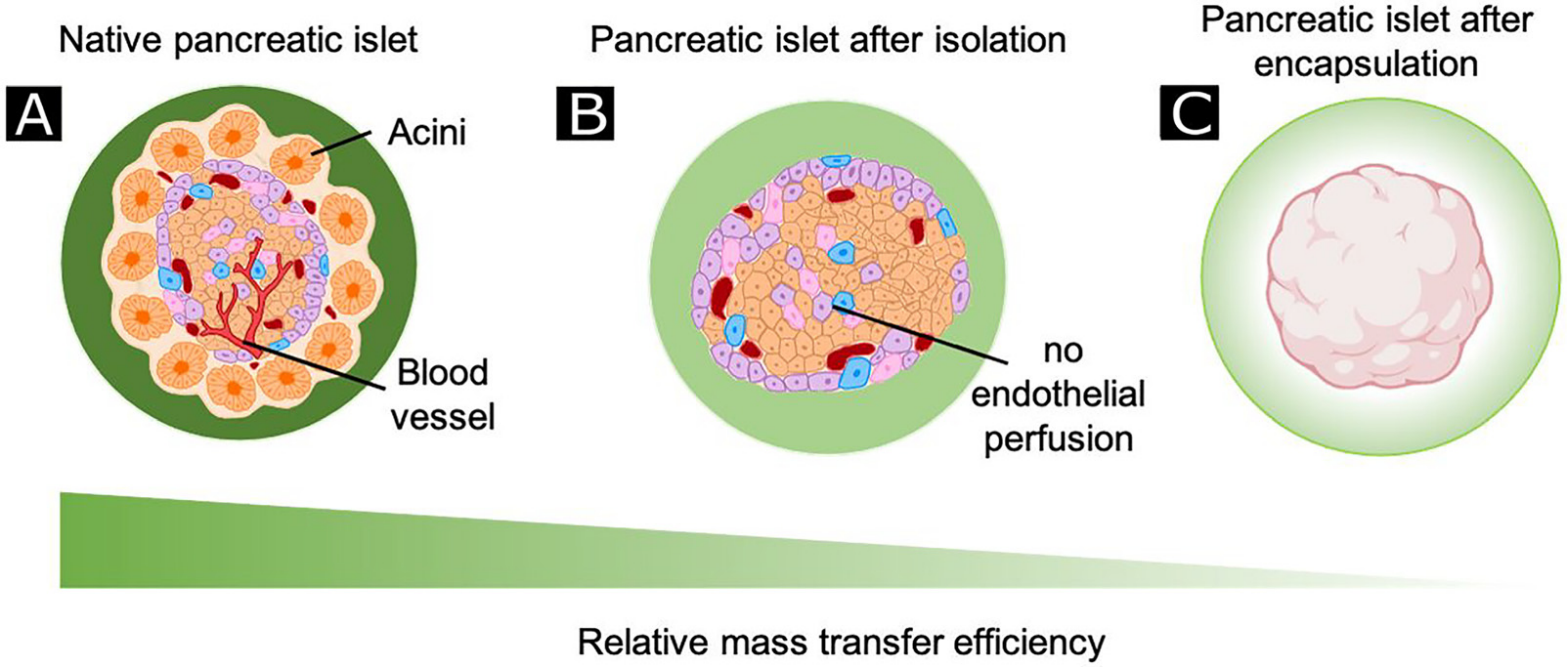
Isolation and encapsulation of islets limit mass transfer. Compared to the native pancreas (a), diffusion dramatically reduced for the majority of cells in islets (especially in the core of the cell mass) as a result of loss of blood perfusion following isolation from the acinar tissue (b). Furthermore, microencapsulation increases the distance of islet cells to the surrounding fluid or blood vessels (c). Dark green represents greater mass transport.

Islet transplantation is known as an effective approach to achieve glycemic control in type I diabetic patients. The successful transplantation depends on critical parameters like compatibility of donor tissue, the efficiency of engraftment, and post-vascularization of the transplanted islets.^45^ Due to the unique anatomical features of the pancreatic islet, which are densely packed large cell aggregates, they require a special vascular network to facilitate the delivery of oxygen and nutrients, as well as rapid insulin release and waste removal.^46^ During the isolation process, encapsulation, and transplantation procedures, the islets lose their vascular network (Fig. 6). The recruitment of ECs is crucial during this recovery period. A recent study demonstrates that indirect co-culturing of brain organoids with patient's own induced pluripotent stem cell (iPSCs)-derived ECs promote organoid vascularization after 3–5 weeks *in vitro* and 2 weeks *in vivo*.^47^ In addition, co-culturing of HUVECs with MSCs has been found to promote vascularization within 7 days.^48^ In another study, co-culturing of human umbilical cord blood EPCs with porcine islet induced secretion of VEGF-A. The authors also reported that they observed early neovascularization in immunodeficient BALB/c nude mice after transplantation.^49^ In light of this information, the use of engineered heterocellular organids is important for the vascularization of larger tissues.

### C. Angiogenic growth factor-based methods

As the main soluble components in neovascularization, GFs can mediate various cellular processes such as cell migration, proliferation, adhesion, differentiation, apoptosis, and ECM synthesis.^50^ In angiogenesis, VEGF, fibroblast growth factor 2 (FGF-2), and angiopoietin 2 (Ang-2) initiate angiogenesis by promoting vessel destabilization, mural cell detachment, and EC activation.^51^ On the other hand, angiopoietin 1 (Ang-1), transforming growth factor β (TGF-β), and PDGFact in the late stages of angiogenesis by promoting vessel maturation, stabilization, and remodeling.^19,52–53^ Since recent engineering strategies for the formation of new blood vessels generally utilize angiogenic GFs, extensive knowledge of their biological functions and their mechanisms of action in neovasculogenesis is essential. GFs can thus be implanted into engineered tissue constructs to leverage their contribution to *in vitro* neovascularization and form intact branched networks. Angiogenic GFs are potent regulators of neovascularization, and their properties are summarized in Table I.^19–22,63–120^ They activate ECs and EPCs; and promote gradient-induced chemotaxis, cell assembly, neovascularization, and maturation. In therapeutic angiogenesis, increased availability of recombinant GFs enabled the administration of pure and soluble GFs. GFs can be injected as either intra-arterial (IA)/intravenous (IV) bolus or directly into the region of ischemia. IA bolus delivery or direct injection of VEGF/FGF in both preclinical and clinical animal studies have successfully restored circulation and promoted angiogenesis in ischemic tissues [Fig. 8(d)].^55,56^ However, the administration of high doses of GFs via bolus injection is associated with undesired side effects. For instance, delivery of VEGF in high concentrations can result in heavy plasma leakage, no-dependent hypotension and edema,^56,57^ tumorigenesis, and uncontrolled vessel formation in undesired locations within the body.^58^

**TABLE I.** The most potent angiogenic growth factors and their mechanism of action.

Growth factor^a^	Ligand/receptor interaction^b^	Angiogenic functions
FGF	FGF-1(aFGF)/FGFR1,FGF-2(bFGF)/FGFR2	Induces EC differentiation, proliferation, migration, adhesion and survival.^63–66^
		Induces the activation, proliferation and migration of other cell types such as EPC and SMCs.^67,68^
		Stimulates angioblast induction.
		Induces vasculogenesis and the formation of immature primary vasculature.^69^
		Stimulates ECM degradation by upregulating the expression of proteases, MMPs and uPA.^63^
		Promotes collateral growth via upregulating PDGFR expression.^70^
		Binds to other cell surface or ECM molecules such as heparin, heparan sulfate proteoglycans (HSPGs) and integrins to enhance their own activity and stability, angiogenic EC response and neovascularization.
VEGF	VEGF/VEGFR1(Flt-1), VEGF/VEGFR2(Flk-1)	Induces vasculogenesis and the formation of immature primary vasculature.^64,69^
		Induces nascent vessel sprouting.^21^
		Induces EC proliferation, migration and survival.^21,71,72^
		Induces EC differentiation and arterial specialization.^71,73^
		Increases vascular permeability and establishes provisional matrix.^74^
		Stimulates protease activity to detach mural cells and degrade the basement membrane for matrix organization and new cell migration.^71^
		Stabilizes vessels by upregulating PDGF-β for mural cell recruitment.^71^
		Stimulates the remodeling of primary vasculature and recruitment of mural cells.^21^
		Inhibits apoptosis and senescence to enhance survival and vessel stability by suppressing p16, p21, p27 and upregulating PI3K/Akt and Bcl2.^22,75^
		Binds to other ECM molecules such as heparin and heparan sulfate proteoglycans (HSPGs) to enhance their own activity and stability, angiogenic EC response and neovascularization; and facilitates co-receptor neuropilin (NRP1) and VEGFR-2 binding.^76^
	VEGF-A_165_/VEGFR2(Flk-1), VEGF-A_165_/NP-1	Enhances EC migration and arterial growth.^77^
	VEGF-C/VEGFR3	Regulates lymphatic vessel development.^78^
	PIGF/VEGFR1	Promotes trophoblast growth, angiogenesis and neovascularization.^79^
		Regulates angiogenic switch by inducing EC proliferation, migration and survival, mobilizing BM-derived cells such as HSCs and recruiting SMCs for vessel stabilization.^80^
PDGF	PDGF-BB/PDGFR-β	Stimulates vessel stabilization and maturation by recruiting MSCs, mural cell progenitors, pericytes and SMCs.^81,82^
		Promotes mural cell proliferation, migration and differentiation.^71^
		Regulates the production of ECM molecules from pericytes to establish basement membrane and ECM of blood vessels; and promote stabilization.^20^
		Contributes to remodeling by causing fibroblasts to secrete collagenases.^20^
		Promotes VEGF expression in vascular SMCs.^83^
	PDGF-AA/PDGFR-α	Regulates angiogenesis by increasing VEGF-A production.^84^
Angiopoietin	Ang-1/Tie-2	Induces vessel stabilization and maturation by inhibiting VEGF activity and plasma leakage, recruiting mural cells, increasing type IV collagen deposition and stimulating EC-cell junction and EC-SMC interactions.^19,69,85–87^
		Regulates EC–EC communication.^87^
		Promotes EC survival by upregulating the expression of survivin, an anti-apoptotic gene through Akt signaling pathway.^88^
		Induces the escape from apoptosis by recruiting ABIN-2 that inhibits NFκB activity.^89^
		Recruits MSCs for their differentiation by TGF-β.^81^
	Ang-1/Tie-2, Ang-1/Tie-2	Regulates tip cell and stalk cell fate determination of ECs (vascular polarity) by upregulating Dll4/Notch signaling.^90^
	Ang-2/Tie-2	Destabilizes vessels by detaching SMCs and relaxing underlying ECM.^91^
		Prevents mural cell recruitment and blocks the activity of Ang-1.^85^
		Induces EC apoptosis and vessel regression in the absence of VEGF.^21^
		Induces EC proliferation and migration; and angiogenic sprouting in the presence of VEGF.^21^
Ephrin	Ephrin-B2/EphB4	Establishes arterial-venous vascular boundary identity.^93^
		Induces vessel sprouting and branching by ECs.^92^
		Stimulates vessel remodeling, stabilization and maturation by recruiting mural cells.^21^
	Ephrin-A1/EphA2	Induces EC migration, proliferation, adhesion and vessel sprouting.^93^
TGF-β	TGF-β1/ALK1	Promotes angiogenesis by inducing EC migration, proliferation and differentiation.^94,95^
		Promotes cell survival and tubule formation *in vitro* by activating PI3K/Akt and Mitogen-activated protein kinase (MAPK) pathways as well as autocrine secretion of TGF-α.^96^
		Upregulates VEGF expression by vascular ECs^97^; and Placental growth factor (PGF)^98^ and bFGF expression by SMCs to enhance angiogenesis.^99^
	TGF-β1/ALK5	Inhibits angiogenesis by hindering EC activity.^94^
		Guides vessel maturation.^71^
	TGF-β1/ TGF-βRII	Induces vessel stabilization and maturation by causing the differentiation of MSCs to mural cells and stimulating ECM deposition.^19,71,81,100^
		Stimulates protease production for vascular remodeling.^71^
HGF	HGF/HGFR	Induces EC proliferation, migration, survival and tubulogenesis.^101^
		Stimulates urokinase secretion by ECs.^102^
		Promotes VEGF expression on VSMCs.^103^
		Enhances VEGF-mediated angiogenesis by ECs.^104^
SDF-1	SDF-1/CXCR4(CD184)	Promotes angiogenesis by recruiting EPCs from BM; and regulating HSC migration and hematopoiesis reconstitution.^105^
		Promotes EC activity, differentiation and tubulogenesis; and inhibits EPC apoptosis.^107,108^
		Promotes vessel stabilization and maturation by recruiting SMC progenitors.^108^
		Promotes vascular remodeling by upregulating metalloproteinases^110^ and downregulating angiostatin.^110,111^
		Regulates the expression of proangiogenic VEGF-A, IL-6, IL-8 and tissue inhibitor of metalloproteinase-2 during vascularization.^110^
SPARC	SPARC^c^	Promotes tubulogenesis of ECs.^112^
		Promotes pericyte recruitment by repressing endoglin-mediated TGF-β1 activity.^113^
		Promotes VEGFR2 activation by blocking anti-angiogenic action of VEGF-A/VEGFR-1 interaction.^114^
		Hinder EC and vSMC activity by inhibiting the activity of VEGFR1, FGF-2 and PDGF.^115–120^

^a^ FGF, fibroblast growth factor; aFGF, acidic fibroblast growth factor; bFGF, basic fibroblast growth factor; VEGF, vascular endothelial growth factor; PIGF, placental growth factor; PDGF, platelet-derived growth factor; Ang, angiopoietin; TGF-β, transforming growth factor β; HGF, hepatocyte growth factor; SDF-1, stromal cell-derived factor 1; SPARC, secreted protein acidic and rich in cysteine; EC, endothelial cell; EPC, endothelial progenitor cell; SMC, smooth muscle cell; MMP, matrix metalloproteinases; uPA, urokinase-type plasminogen activator.

^b^ FGFR, fibroblast growth factor receptor; VEGFR, vascular endothelial growth factor receptor; NP-1, neuropilin 1; PDGFR, platelet-derived growth factor; Tie-2, tyrosine kinase with Ig and EGF (epidermal growth factor) homology domains; Eph, ephrin receptor; ALK, anaplastic lymphoma kinase; TGF-βR, transforming growth factor β receptor; HGFR, hepatocyte growth factor receptor; CXCR4, C-X-C chemokine receptor type 4.

^c^ Uncharacterized receptor.

Moreover, GFs are highly unstable *in vivo*, which makes it challenging to maintain a constant dose of recombinant protein over the required period at the ischemic site. In another study, Waters and others developed an injectable system consisting of a therapeutic moiety (secretome), gelatin, and Laponite^®^.^59^ The authors used this biocompatible and injectable system to implant into peri-infarct myocardium in rats. Both *in vitro* and *in vivo* analyses revealed the pro-angiogenic activity of the construct. A significant increase in capillary density was observed with a non-significant immune response. Therefore, biomaterial-based strategies (e.g., controlled delivery of encapsulated GF within biopolymeric matrices or nanoparticles) have been preferred over the delivery of pure GFs at high concentrations. GFs can be delivered in a sustained manner using purpose-built biomaterial scaffolds, which will be explained in Sec. II C 1 in detail. One other option is the delivery of genes encoding GFs in vectors. For example, plasmids carrying VEGF and hepatocyte growth factor (HGF) genes have been used to induce angiogenesis in rat models of myocardial infarction and diabetic hind limb ischemia, respectively.^62,63^ A third alternative is the transplantation of cells that have been genetically modified to overexpress GFs, which provides sustained GF release. In one example, MSCs expressing VEGF were introduced into infarcted myocardium; they restored the heart function and enhanced angiogenesis along with blood perfusion [Figs. 8(a)–8(c)].^62^ However, since it is not possible to regulate gene expression timing and quantity in transfected cells, controlled and continuous release of GFs is safer for clinical applications.

#### 1. Growth factor delivery strategies

**FIG. 7.**
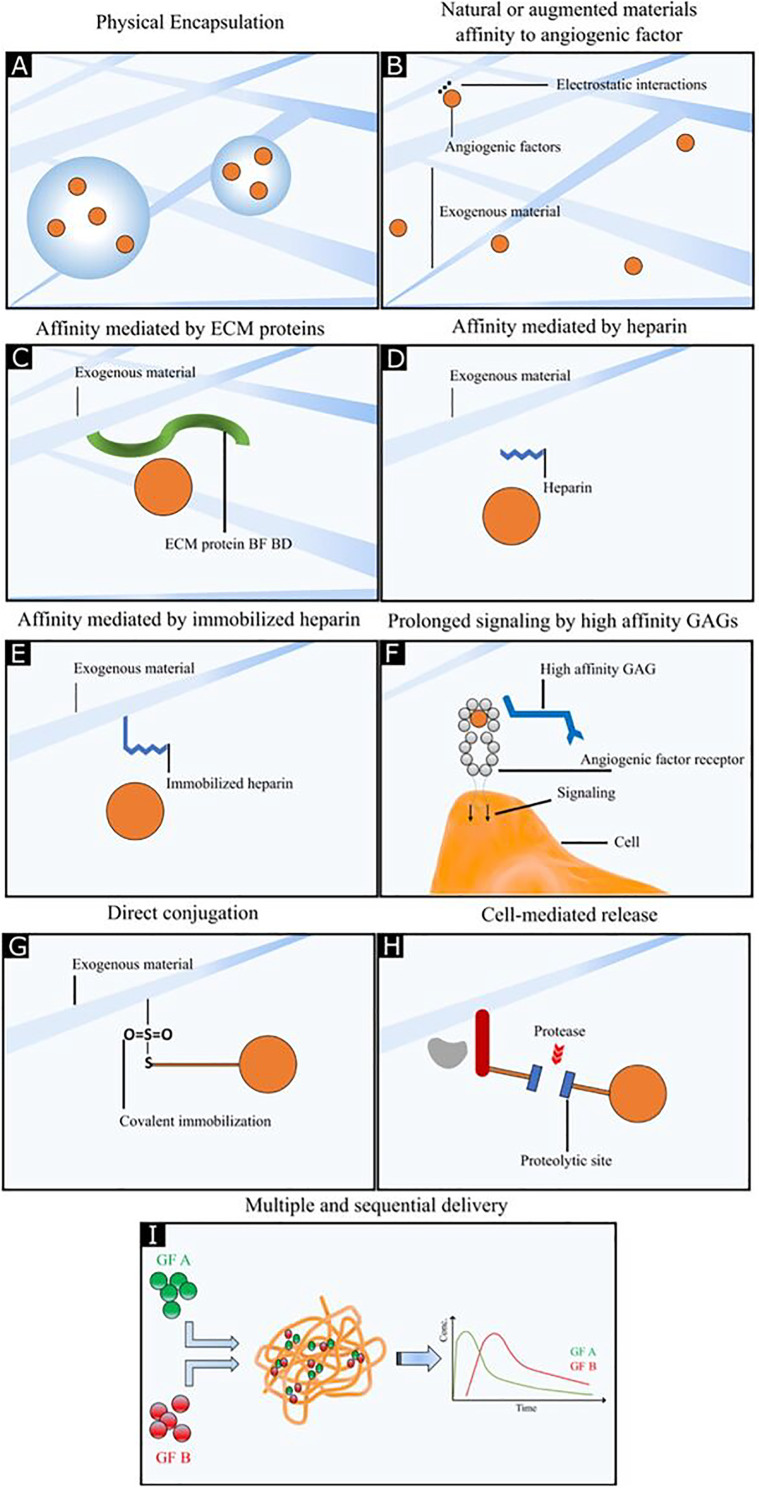
Angiogenic growth factor delivery strategies. (a) Physical encapsulation of growth factors which provides sustained and local GF's release at the target site with better retention of biological activity. (b) Natural or augmented materials affinity to angiogenic factor in which sustained and localized release are obtained by use of ionic complexation between oppositely charged groups on GFs and biomaterial scaffolds. Binding interaction through (c) affinity mediated by ECM proteins. (d) Affinity mediated by heparin. (e) Affinity mediated by immobilized heparin. (f) Prolonged signaling by high-affinity glycosaminoglycans (GAGs) prolongs the retention of GFs within the scaffold, increases their stability, and protects them from denaturation by heat, inactivation at acidic pH inactivation, and proteolytic degradation. (g) Direct conjugation provides more prolonged GF retention and release. (h) The cell-mediated release makes the system responsive to environmental stimuli (such as pH, temperature, proteolytic cleavage site, ions, light, drug, magnetic, and electric field) which provide temporal control over GF. (i) Multiple and sequential delivery provide better recapitulation of an *in vivo* microenvironment where more than one factor has involved the process.

Polymeric biomaterial scaffolds enable spatial and temporal control over GF availability, release, and biological activity. Delivery of GFs from biomaterials avoids their rapid clearance from the target site and provides continuous and prolonged release with reduced adverse effects (e.g., cytotoxicity, hypotension, excessive vascular leakage, etc.). As a result, biomaterial scaffolds carrying GFs provide the best route for GF delivery compared to bolus injections or systemic administrations. This section will cover the following modes of GF delivery by biopolymeric matrices for therapeutic angiogenesis: physical encapsulation of GFs, ionic complexation, release by GF-binding molecules, immobilization of GFs including covalent conjugation, on-demand GF delivery, and delivery of multiple GFs. The schematic representation of these GFs modes is illustrated in Fig. 7 and details are enlisted in Table II.^93,121,125,129–135^

**TABLE II.** Examples of growth factors delivery strategies for therapeutic angiogenesis.

Delivery strategy	Growth factor	Methodology	Results
Physical encapsulation	FGF-2	Light-induced crosslinkable chitosan hydrogels were loaded with bFGF	Encapsulation led to the sustained release of greater bFGF content through *in vivo* degradation; and achieved augmented wound healing and microvessel formation in diabetic mice^121^
Microsphere and nanoparticle-mediated delivery	VEGF	PLGA microspheres encapsulating VEGF has been incorporated into dextran hydrogels to form composite hydrogels	
			This hydrogel increased VEGF receptor Flk-1 approximately 20-fold and vascular differentiation of hESCs more than embryoid body cultures^125^
Ionic complexation	SDF1-α	SDF1-α formed ionic complexation with anionic succinylated gelatin hydrogels	Ionic complexation led to increased GF retention, prolonged release and augmented angiogenesis after implantation^129^
Immobilized GAG and GF-binding domain-mediated delivery	FGF-2	FGF-2 was loaded into collagen matrices that covalently incorporates heparan sulfate, and matrices were implanted to rat	Heparan sulfate coupling increased binding capacity and retention of FGF-2 threefold and resulted in its sustained and prolonged FGF-2 release *in vitro*, and augmented neovascularization *in vivo*^130^
Covalent conjugation/immobilization	Ephrin-A1	Ephrin-A1 was covalently conjugated to PEGDA hydrogels	Covalent conjugation enhanced HUVEC adhesion, tubulogenesis; and stimulated stabilization by increased depositions of ECM proteins (laminin and collagen IV) *in vitro* and formed enlarged and highly branched microvasculature with higher vessel density and lower vessel diameter *in vivo*^93^
Spatiotemporal delivery	VEGF	End-functionalized PEG hydrogels were cross-linked with MMP substrate covalently conjugated with thiol-containing the tripeptide Arg-Gly-Asp (RGD) and VEGF through Michael-type addition reaction	Hydrogel system enhanced MMP secretion by activating angiogenic cells such as HUVECs and VSMCs; and cleaved cross-linking MMP substrate. MMP-mediated degradation caused matrix-bound VEGF liberation, resulting in enhanced, long-term and controlled angiogenesis with mature microvasculature stabilized by SMCs^131^
Simultaneous delivery of non-covalently conjugated multiple GFs	VEGF, Ang-1, SDF-1, IGF	GFs were co-immobilized in dextran hydrogels and the system was compared with the groups with fewer factors	Simultaneous delivery of 4 factors led to greater proangiogenic synergistic effect that resulted in functional microvasculature with increased number of larger and more mature blood vessel formation than hydrogels immobilized with individual GF^132^
Simultaneous delivery of covalently conjugated multiple GFs	VEGF, Ang-1	3D collagen scaffolds were co-immobilized with VEGF and Ang-1 via EDC chemistry	Dual delivery led to more enhanced EC proliferation, attachment and tubulogenesis *in vitro*; and more mature and stable vessels and increased hemoglobin concentration indicating augmented angiogenesis with enhanced vessel density and proper connection to host circulation in CAM assay *in vivo* than their soluble controls and single GF immobilization groups that lack proper vascularization^133^
Sequential delivery of multiple GFs	VEGF, PDGF, Ang-1, Ang-2	Scaffolds formed from PLGA microspheres through gas foaming were loaded with VEGF, PDGF, Ang-1 and Ang-2	Sequential delayed delivery of early and late angiogenic factors led to enhanced EC activity, pericyte detachment mediated-vessel disruption and new vessel sprouting by VEGF and Ang-2; and augmented microvessel remodeling, density, stabilization and maturation by PDGF and Ang-1 without inhibiting each other's activity compared to simultaneous delivery of all factors where late GFs inhibit the actions of early GFs^135^
Spatiotemporal delivery of multiple GFs	VEGF, PDGF-BB	Bilayer PLGA scaffolds was loaded with only VEGF in one spatial zone and both VEGF and PDGF-BB in nearby zone for a sequential delivery	Spatiotemporal delivery resulted in the significant augmentation of maturity and vessel size^135^

##### a. Physical encapsulation of GFs

**FIG. 8.**
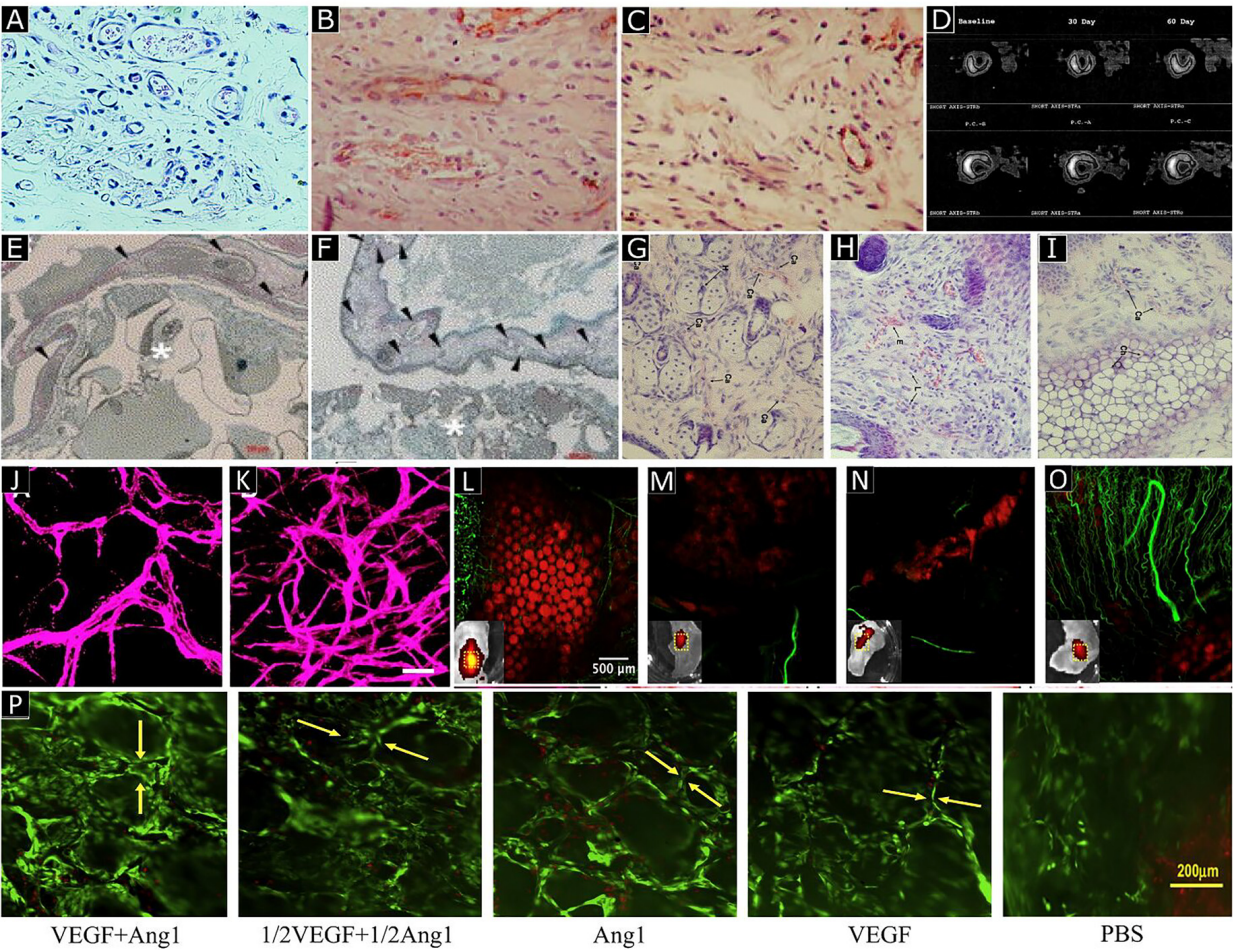
Angioma formation and angiogenesis in rats treated with phVEGF (a). Reprinted with permission from Schawrz *et al.*, J. Am. Coll. Cardiol. **35,** 1323 (2000). Copyright 2000 Elsevier. Enhanced angiogenesis by VEGF-expressing Marrow MSCs (reddish-brown). An increased number of vessels were observed in groups treated with gene delivery (b) compared to the control group (c). Reprinted with permission from Yang *et al.*, Cardiology **107**, 17 (2007). Copyright 2007 Karger Publishers, Basel, Switzerland. Serial Single-photon emission computed tomography (SPECT) myocardial perfusion images showed the bolus delivery of rhVEGF restored circulation and promoted angiogenesis in ischemic tissues (D). Reprinted with permission from Henry *et al.*, Am. Heart. J. **142**, 872 (2001). Copyright 2001 Elsevier. VEGF encapsulated in PLA scaffolds present in CAM. Histological analyses showed increased vessel number (arrowheads) in the PLA-VEGF scaffold (f) compared to control (e). Reprinted with permission from Kanczler *et al.*, Biochem. Biophys. Res. Commun. **352**,” 135 (2007). Copyright 2007 Elsevier. Hematoxylin and eosin staining images of mice ear tissue, heparin-HA-VEGF hydrogel showed greater neovascularization with well-defined vascular borders (g) compared to HA-VEGF specimen (h) and control (I). Reprinted with permission from Pike *et al.*, Biomaterials **27**, 5242 (2006). Copyright 2006 Elsevier. The covalent conjugation of ephrinA1 and PDGF to PEGDA hydrogels showed greater neovascularization (k) compared to PDGF-BB alone conjugation (j). Reprinted with permission from Saik *et al.*, Biomacromolecules **12**(7), 2715–2722 (2011). Copyright 2011 American Chemical Society. Vascularization analysis through fluorescent images perfused with lectin (green) to label vasculature after 14 days of implantation in mice in non-degradable microgel with VEGF (l), degradable microgel without VEGF (m), degradable microgel with a bolus injection of VEGF (n) and degradable microgel with VEGF (o). Reprinted with permission from Foster *et al.*, Biomaterials **113**,170 (2017). Copyright 2017 Elsevier. Fluorescence live (green)/dead (red) image of H5V cells on collagen scaffolds showed greater vessel formation (yellow arrows) in co-immobilized growth factor groups compared to single growth factor groups and control (p). Reprinted with permission from Chiu *et al.*, Biomaterials **31**, 226 (2010). Copyright 2010 Elsevier.

Physical encapsulation, which involves the entrapment or loading of GFs in a biomaterial-based scaffold, is the simplest method for GF delivery in biomaterial scaffolds [Fig. 7(a)]. Encapsulation provides sustained and local GF's release at the target site with better retention of biological activity and reduces the spike in released GF concentration compared to bolus injection. When chitosan hydrogels were loaded with bFGF in diabetic mice, most of its bFGF content was released over an extended period (10–14 days) through *in vivo* degradation of chitosan; and augmented wound healing and microvessel formation was observed.^121^ Many natural and synthetic biomaterials [e.g., fibrin, hyaluronic acid (HA), gelatin, polyethylene glycol (PEG), poly(lactide-co-glycolide) (PLGA), etc.] have been used to encapsulate proangiogenic GFs by mixing GF and polymer prior to solidification or gelation; these biomaterials exhibit varying angiogenic responses depending on scaffold properties such as porosity, water content, density, and fabrication protocol. For example, when VEGF was encapsulated in hydrophobic degradable PLGA microspheres prior to fabrication into PLGA scaffolds, this approach led to prolonged and sustained GF release, and thus higher local angiogenesis *in vitro* and *in vivo* compared to VEGF that was directly incorporated into PLGA scaffolds.^122^ Encapsulated VEGF in Polylactic acid (PLA) scaffolds was shown to enhance capillary formation by HUVECs *in vitro* and augment microvasculature development *in vivo* [Figs. 8(e) and 8(f)].^125^ The release rate and period of encapsulated GF can also be controlled by adjusting polymer rigidity, degradation rate, and cross-linking density as slow degradation leads to prolonged GF release and vice versa. Compared to other GF delivery methods, physical encapsulation better preserves the loaded GFs, but their release profile remains unpredictable, and the amount of loaded GF limited.

Another strategy for physical encapsulation of GFs includes the use of microspheres and nanospheres, which are micrometers- and nanometer-sized spherical particles with a high surface-to-volume ratio that can be fabricated from natural or synthetic polymers. Precise control over the size and degradation rate of these particles enables the timely release of GFs with precise kinetics as the size of these nano- and microparticles determines their surface-to-volume ratio, which affects GF release rate. The composition of particles can also be modified depending on the specific target tissue so that GF release can occur only when these particles encounter proteins or cells in the target area. These features prevent diffusion of GFs out of the target zone upon degradation. The small diameters of nano- and microspheres (which vary between 50–700 nm and 10–100 *μ*m, respectively) enable them to infiltrate cells easily and promote angiogenesis.^50^ In one study, calcium alginate microspheres loaded with VEGF exhibited localized, and prolonged GF release along with enhanced microvessel formation and development when transplanted in rats.^124^ Microparticles may also be incorporated into biomaterial scaffolds to achieve more localized GF release. For instance, PLGA microspheres encapsulating VEGF have been incorporated into the dextran hydrogel backbone to form composite hydrogels. This functionalized hydrogel was shown to increase the amount of hESCs expressing the VEGF receptor Flk-1 approximately 20 fold, while also improving their vascular differentiation compared to embryoid body cultures.^125^ Microspheres can also enable the formation of layered biomaterial scaffolds with different GF concentration gradients. For example, a multilayer scaffold was formed by compacting a series of microsphere layers. Each layer containing varying concentrations of VEGF resulted in the formation of a VEGF gradient across the thickness. After implantation into the ischemic limb of the mouse, it induced a similar appearance to healthy native hindlimb by increasing microvasculature formation, vessel density, and perfusion.^126^ In general, nanoparticles are promising vehicles of GF delivery for stimulation of angiogenesis as they can be internalized by cells efficiently and quickly penetrate organelles.

##### b. Ionic complexation

Another simple method to entrap GFs and achieve their sustained and localized release is the use of ionic complexation between oppositely charged groups on GFs and biomaterial scaffolds [Fig. 7(b)]. Many positively charged GFs (e.g., bFGF) with surface-exposed lysine and arginine residues can interact with negatively charged polymers, thereby leading to a slower initial release of GFs. For instance, bFGF complexed with acidic gelatin hydrogels with an isoelectric point (pI) of 5.0 led to augmented angiogenesis as a result of slow, prolonged, and sustained bFGF release whereas bFGF ionically complexed with basic hydrogels (which also have a net positive charge at neutral pH, thereby repelling bFGF) resulted in burst release and short-lived angiogenesis.^127^ Consistent with this observation, VEGF complexed with PLGA microspheres containing free acidic end groups resulted in a more potent and slower GF release than that achieved with microspheres without acidic groups.^128^ Therefore, despite its promising use in sustained and localized GF release, ionic complexation also suffers from mentioned drawbacks where this technique cannot be applied to all kinds of polymers.

##### c. Controlled release by growth factor-binding molecules

A more potent method to promote angiogenesis is the modification of biomaterials with glycosaminoglycans (GAGs, e.g., heparin, heparan sulfates, hyaluronic acid) or active GF-binding domains of ECM proteins. Many angiogenic GFs such as FGF-2, vascular endothelial growth factor A (VEGF-A), and PDGF-BB naturally interact with the heparin and heparan sulfate polymers in the ECM. Functionalization of biomaterials with heparin and heparan sulfate provides binding sites for these GFs [Figs. 7(d) and 7(e)]. This binding interaction prolongs the retention of GFs within the scaffold, increases their stability, and protects them from denaturation by heat, inactivation at acidic pH inactivation, and proteolytic degradation.^17,136^ Stabilized GFs within biological scaffolds remain active and are released locally in a continuous fashion to enhance vascular cell migration, proliferation, and neovascularization. For instance, collagen matrices covalently conjugated with heparin by 1-ethyl-3-(3-dimethylaminopropyl)carbodiimide (EDC) and N-hydroxysuccinimide (NHS) chemistry led to enhanced EC proliferation and angiogenesis with or even without VEGF *in vitro* and *in vivo*. Thus, immobilized heparin significantly enhanced the activity of endogenous GFs.^137^ In another study, the effect of heparin concentration and the use of GAG carriers on GF release were evaluated by using heparin-containing hyaluronan hydrogels loaded with either bFGF or VEGF. For this, hyaluronan, gelatin, and heparin were modified with thiol groups, and gelation was achieved through thiol-ene click chemistry in the presence of PEGDA. Excess heparin was shown to decrease GF release, gel stability, and the rate of cross-linking, and optimum heparin concentration was determined to be less than 1% of gel content (approximately 0.3% by weight). At this concentration, heparin addition to hyaluronan prolonged GF retention without the loss of GF activity, while extending the period of GF release and microvasculature growth up to 28 days *in vivo* [Figs. 8(g)–8(i)].^138^ Heparin can also inhibit aggregation of GFs, which can occur due to non-specific interactions between neighboring GFs at high concentrations.^139^ For example, heparin conjugation to polymeric micelles carrying bFGF resulted in almost complete release of bFGF from the matrix membrane compared to the low release rate (20%) from heparin-free matrices, in which intermolecular aggregation prevented the passage of GFs through the matrix membrane.^140^ Another approach is to use GF-binding domains of EC proteins instead of immobilized heparin to provide binding sites for GFs [Fig. 7(f)]. For instance, fibrin and fibrinogen naturally contain a heparin-binding domain (heparin-binding domain II) for PDGF/VEGF, FGF, and TGF. When this heparin-binding domain was immobilized to PEG matrices, it completely mimicked the GF-binding capacity of fibrin to induce angiogenesis in a diabetic mouse *in vivo*.^141^ Finally, GF-binding peptides derived from GF receptors can be used for the same purpose. For example, VEGF-binding peptide was derived from VEGFR2 and was covalently conjugated to hydrogel microspheres, which enabled controlled VEGFA release and the regulation of HUVEC proliferation.^142^

##### d. Immobilization of growth factors

Immobilization or conjugation of GFs to biomatrics is a powerful method to enhance neovascularization in engineered tissues. GFs can be immobilized by either non-covalent interactions (e.g., ionic interactions between oppositely charged groups on GFs and biomaterial scaffold) or by covalent coupling. They can also be immobilized indirectly via GAGs and GF-binding domains of ECM proteins (e.g., fibrin, fibronectin, etc.) that serve as intermediaries for binding GFs, as explained in Sec. II C 1 c. A swelling- or diffusion-based mechanism generally controls the release of non-covalently immobilized GFs. Covalent conjugation involves the direct chemical coupling of intact GFs or GF domains to biomatrices. This method leads to more prolonged GF retention and release compared to techniques explained in Sec II C 1, as well as a significant increase in GF stability by protecting against proteolytic cleavage and heat inactivation [Fig. 7(g)]. GFs can be covalently conjugated to biomaterial scaffolds using photochemical grafting, Michael addition reactions, or carbodiimide (EDC) chemistry.^143^ Covalent conjugation of GFs provides several advantages. First, GFs tethered to biological scaffold continue to activate their receptors due to the reduction in GF degradation rate as well as GF internalization by cells compared to non-covalent conjugation methods that lead to GF internalization.^144^ Second, the direct coupling of GFs to biomatrices eliminates GF aggregation and contributes to their sustained release. Third, this method does not require the use of intermediate molecules acting as GF-binding sites such as endogenous heparin, heparan sulfate, and ECM proteins. The release profile of covalently immobilized GFs can be tuned by the following: (a) the rate of matrix degradation, which in turn, can be controlled by adjusting the degree of cross-linking, degradation rate, pore size, and porosity, as explained previously; and (b) the use of enzymatically or chemically controlled mechanisms (e.g., pH, temperature, MMP-cleavage site incorporation) for GF release on demand. Various covalent immobilization schemes have been successfully tested for therapeutic angiogenesis. In one study, ephrin-A1 that was covalently conjugated to PEGDA hydrogels was shown to enhance angiogenic ephrinA1–EphA2 interaction, cell adhesion, tubule formation by HUVECs, and tubule stabilization by increased deposition of ECM proteins (laminin and collagen IV) *in vitro*. When these ephrin-A-conjugated hydrogels were implanted into mouse cornea, an enlarged and highly branched microvasculature network formed with higher vessel density and lower vessel diameter. These results indicated that a more effective vascularized tissue network stabilized through ECM-deposition can be generated by immobilizing ephrinA1 [Figs. 8(j) and 8(k)].^93^ Proangiogenic GFs can also be covalently coupled to scaffolds to create concentration gradients. In this method, different amounts of GFs are covalently immobilized at different locations within a scaffold to create a gradient, thereby recapitulating *in vivo* chemotaxis to direct EC migration, proliferation, and neovascularization. Additional modifications with adhesive ligands such as the RGD tripeptide can further improve cell adhesion and angiogenesis. For instance, PEG hydrogels were covalently immobilized with a bFGF gradient, and cell adhesive RGD peptide and concentration gradient was established by locking hydrogel prepolymer solution upon UV-mediated photopolymerization. This method has been shown to increase vascular smooth muscle cell (VSMC) migration (∼15%) and proliferation (∼41%). The chemotactic activity was recapitulated as SMCs migrated differentially across the patterned bFGF gradient. Therefore, higher retention of bFGF and the continued presence of the GF concentration gradient enabled better VSMC chemotaxis, which is important during late angiogenesis, where VSMCs are recruited *in vivo* to stabilize nascent vessels. Furthermore, the addition of the adhesive RGD ligand further augmented angiogenic response which was already bolstered by the immobilized bFGF gradient.^145^ Although site-specific conjugation of GFs enables more potent GF activity and delivery, there are possible limitations such as loss of GF activity due to denaturation during conjugation reactions. Another drawback is the lack of precision in determining the coupling site on the surface of GFs since the reactive group (e.g., primary amino group on the lysine side chain) may appear at multiple locations on the protein surface. Covalent conjugation at undesired locations may reduce the biological activity of the GF after coupling (e.g., due to steric hindrance) or eliminate it (e.g., due to occlusion of the binding site).^144^

##### e. On-demand delivery

The main goal of using growth factor delivery systems is to achieve localized and controlled GF release. Although GF encapsulation in or conjugation to scaffolds are powerful methods to achieve this aim, additional modification of matrices with components responsive to environmental stimuli can provide temporal control over GF release and make it more localized. The main triggers for GF release in *in vivo* conditions such as pH, temperature, proteolytic cleavage site, ions, light, drug, magnetic and electric field are critical for a successful design [Fig. 7(h)]. Change in local pH is one of the most widely used methods to trigger and control GF release kinetics at a target site. Hydrogels, which are highly stable at physiological pH, reversibly lose their stability upon a decrease in pH due to protonation of functional groups triggering the release of their GF content. Also, temperature-responsive polymers such as poly(N-iso-propylacrylamide) (pNIPAAM) can undergo reversible gelation with low cytotoxicity upon heating (from room temperatures to physiological temperatures), thereby enabling localized time-dependent GF release.^146^ Given the acidic microenvironment of the ischemic myocardium, temperature- and pH-sensitive hydrogels have been utilized to provide spatiotemporal control over bFGF delivery. In one example, poly(N-isopropylacrylamide-co-propylacrylic acid-co-butyl acrylate) (p[NIPAAm-co-PAA-co-BA]), a pH- and temperature-sensitive copolymer, was used to generate an injectable hydrogel. The polymer remained liquid at pH 7.4 and room temperature and formed a solid gel at pH 6.8 and 37 °C. After injection of polymer with bFGF into infarcted rat heart, the polymer formed a gel under the acidic conditions of the ischemic myocardium. The gel demonstrated prolonged and local bFGF retention (tenfold higher over seven days post-injection compared to control groups). While, controlled and sustained bFGF release, augmented microvessel density and blood circulation (40% and twofold increase, respectively, 28 days post-injection compared to control groups) was also observed. The hydrogel dissolved completely once the infarcted tissue was restored and returned to its normal physiological pH. This system exhibited sustained and local delivery of bFGF while improving angiogenesis, blood flow, and, ultimately, cardiac function.^147^

The most common method to trigger the on-demand release of GFs and promote angiogenesis is the use of proteases in matrices that include protease-sensitive oligopeptides. The most widely used group of proteases in angiogenic GF delivery studies are matrix metalloproteinases (MMPs), particularly MMP-2. Incorporation of cell-adhesive ligands into the scaffold, in turn, promotes EC invasion of the scaffold surface. Upon cellular demand, MMPs secreted by these invading cells cleave these protease-sensitive sites, expose the vascular or mural cells to the underlying scaffold coated ischemic sites, thereby providing localized and controlled GF release. In an MMP-mediated GF delivery study, the cell-adhesive tripeptide RGD and VEGF were covalently coupled to PEG macromers via Michael addition reaction. Next, MMP-2-sensitive oligopeptides were incorporated into the polymer backbone of this functionalized PEG hydrogel. HUVEC adhesion and proliferation were enhanced by RGD and VEGF; MMP-2 secretion was upregulated by HUVEC migration and VEGF activity, leading to hydrogel degradation along with localized and sustained VEGF release. TGF-β1 was also encapsulated to regulate MMP-2 activity and alleviate a possible inhibitory effect of TIMP-1 on MMP-2. In the end, controlled and localized MMP-2-mediated-release of VEGF and enhanced EC activity were observed *in vitro*.^148^ Similarly, in an *in vivo* study,^149^ microfluidics-based polymerization was carried out to synthesize an injectable PEG-based microgel of defined size. Crosslinking was performed by using a degradable peptide. Covalently tethered protein was released from the microgel network in response to local protease in mice. The release rate was tuned and optimized by using different ratios of non-degradable (Dithiothreitol (DTT)-based) and degradable crosslinker (VPM-based crosslinker). The controlled release of VEGF from degradable microgel resulted in enhanced vascularization as compared to bolus injection or control (microgel with no VEGF) [Figs. 8(l)–8(o)]. In another *in vivo* study,^131^ PEG hydrogels functionalized with divinylsulfone were covalently conjugated to thiol-containing RGD and VEGF through a Michael addition reaction. Invading ECs locally, remodeled the biomaterial by secreting MMPs, which cleaved the cross-linking MMP-sensitive sequences within the material. MMP-mediated degradation allowed further cell invasion into the matrix and liberated VEGF coupled initially to the matrix. When VEGF-containing hydrogels were placed on the top of a chick chorioallantoic membrane (CAM) membrane, the resulting MMP-mediated VEGF release and enhanced angiogenesis were observed only in the contact area of the CAM and graft membrane. In addition, all the formed microvasculature was mature, stabilized by SMCs with no presence of primitive or undesired vessels. These results have confirmed the success of this approach in inducing spatiotemporal and prolonged neovascularization at the target site. Other examples of triggerable GF release include controlled release of TGF-β1 from photodegradable hydrogels upon light exposure,^150^ the release of protein content from hydrogels with ion-binding proteins upon Ca^2+^ treatment,^151^ controlled TGF-β1 release from magnetic field-sensitive alginate ferrogels formed from iron oxide nanoparticles through ionic cross-linking^152^ and the use of electric field-sensitive polymer matrices. However, the translation of these trigger systems to a clinical setting may be limited or impractical since these are highly laborious, expensive, and involve a complex operation. Further optimization of these systems in terms of light, electric, or magnetic field dose according to standards is needed for translation to the clinics.

##### f. Delivery of multiple growth factors

Although localized and controlled delivery of a single GF helps to recapitulate angiogenesis *in vitro*, the need for different GFs at different steps of angiogenesis necessitates the delivery of multiple proangiogenic GFs [Fig. 7(i)]. In the natural microenvironment, each proangiogenic GF performs a specific function in coordination with other GFs at various steps of angiogenesis. For instance, VEGF, FGF, Ang-2, and Eph-B2 function in early angiogenesis, while PDGF-BB, Ang-1, and TGF-β1 are upregulated in late angiogenesis.^19,21,69,71^ Therefore, researchers can mimic the coordinated interactions of these different GFs by delivering multiple GFs simultaneously or sequentially at specific concentrations and locations to obtain a stable and mature microvasculature.

The simplest way to deliver multiple GFs is to release them from a scaffold simultaneously. For example, collagen scaffolds modified with heparin enabled the binding of FGF-2 and VEGF; these scaffolds led to higher blood vessel density and vessel maturation by SMCs when compared to ones containing a single GF. The combination of two proangiogenic GF thus displayed a synergistic effect on neovascularization and rapidly formed robust and mature microvessels.^153^ In a similar study, the tetrapeptide Arg-Gly-Asp-Ser (RGDS)-modified PEGDA hydrogels were both covalently conjugated with ephrinA1 and encapsulated with PDGF. When implanted into mouse cornea, these hydrogels provided enhanced neovascularization with higher vessel density, reduced mean diameter, and a more complex branched network than hydrogels containing only PDGF.^93^ In another study, when VEGF and Ang-1 were covalently coupled to 3D collagen scaffolds using carbodiimide chemistry, they augmented EC proliferation, attachment, and tubule formation *in vitro*. They also highly enhanced vessel density and increased hemoglobin concentration (which indicated more successful angiogenesis). When they were implanted into the chick chorioallantoic membrane (CAM), proper connection to host circulation in CAM assay *in vivo* was observed. These results proved the superiority of dual covalent GF conjugation in inducing angiogenesis over collagen scaffolds, which encapsulated soluble GFs or contained a single GF as the latter ones lacked proper vascularization. Moreover, newly formed blood vessels were more mature and stable in experimental groups with both VEGF and Ang-1. VEGF promoted EC proliferation, migration, and increased vascular permeability for better EC invasion and vessel sprouting, whereas Ang-1 acted as an anti-permeability factor by reducing VEGF action and regulating vessel remodeling, stabilization, and maturation through mural cell recruitment. This was the first study to immobilize Ang-1 and demonstrate the successful induction of angiogenesis by co-immobilized VEGF and Ang-1 [Fig. 8(p)].^133^ More than two GFs can also be delivered simultaneously. For instance, when compared to single GF delivery, co-immobilization of VEGF, Ang-1, SDF-1, and insulin-like growth factor (IGF) in dextran hydrogels was shown to induce rapid and robust neovascularization. Here, VEGF initiated functional development, SDF-1 and IGF increased the size and number of vessels and Ang-1 induced vessel maturation. As a result, compared to hydrogels carrying a single GF, the delivery of multiple GFs could confer a synergistic proangiogenic effect that resulted in functional microvasculature with an increased number of larger and more mature blood vessels.^132^ Another potent approach involves sequential delivery of multiple GFs, which more faithfully mimics angiogenesis by enabling different release kinetics for each GF. Since each proangiogenic GF is active at different stages of angiogenesis, proangiogenic GFs should be delivered sequentially to prevent undesired cross-reactivity and achieve more natural and potent neovascularization. The first study in which multiple GFs were delivered sequentially, utilized PLGA matrices formed by mixing two scaffolds; one synthesized from PLGA particles mixed with VEGF, and the other from PLGA microspheres pre-encapsulated with PDGF-BB. As a result of the use of differently processed scaffolds, VEGF showed rapid, while PDGF-BB exhibited a slow-release rate. Sustained and localized delivery of these two GFs initiated the formation of a large number of blood vessels and induced their maturation compared to their single or simultaneous delivery *in vivo* since VEGF initiates angiogenesis and PDGF-BB recruits mural cells for maturation afterward.^154^ In another study, alginate hydrogels containing VEGF and PDGF-BB were shown to recapitulate natural angiogenesis by successfully providing sequential release of VEGF-A followed by PDGF-BB with different release kinetics. In this system, the difference in affinities of PDGF-BB and VEGF to alginate led to distinct release kinetics for each GF. Since the release of PDGF-BB was delayed and occurred more slowly after the initial three-day-period release of VEGF, PDGF-BB became active only in the late stages of angiogenesis. First, released VEGF initiated angiogenesis, and then, the late release of PDGF-BB stabilized the nascent vessels by recruiting SMCs and enhancing VEGF-induced proliferation of SMCs. This system led to more potent vascularization with more mature and stable vessels lined with SMCs. Consequently, this system improved myocardial function with higher tissue perfusion compared to a single factor delivery in aortic ring model *in vitro* and *in vivo*.^135^

The sequential delivery of more than two factors has also been demonstrated. For example, scaffolds formed from PLGA microspheres through gas foaming were utilized to sequentially deliver VEGF, PDGF, Ang-1, and Ang-2. Coordinated delivery of early angiogenic VEGF and Ang-2 enhanced EC activity, vessel disruption from pericyte detachment, and new vessel sprouting, while subsequent delivery of late angiogenic PDGF and Ang-1 increased density and induced microvessel remodeling, stabilization and maturation without inhibiting vessel sprouting. A regimented delivery of these four GFs yielded superior results than their simultaneous delivery, suggesting that delayed release of late angiogenic factors also improved the function of early proangiogenic factors for better angiogenesis. In the end, rapid delivery of VEGF and Ang-2, along with the delayed release of PDGF and Ang-1, resulted in greater neovascularization, yielding a higher number of mature vessels with larger diameters *in vitro* and *in vivo*.^134^

## III. CLINICAL TRANSLATION OF ENGINEERED TISSUES

The goal of tissue engineering and regenerative medicine is the clinical translation of developed tissues or organs in laboratories. Until now, a fully developed vascularized organ in a laboratory is far proven yet. However, continuous advancement in research and technology has enlightened the hope for the replacement of diseased and damaged tissues or at least their repair. Functionalized biomaterials are being explored for targeted tissue engineering applications. Intense research is going on for developing new procedures and methods to build efficient scaffolds that can serve the purpose of end-use applications. Cells in an engineered tissue should be within a diffusion distance of 100–200 *μ*m for sufficient nutrient and gas exchange; this fact poses a significant challenge to scientists and engineers in developing a fully vascularized tissue. Typically, when tissue is implanted in the patient, blood vessels from the host tissue invade to develop such a vascular network. This ingrowth, however, is usually very limited and takes a long time, thereby resulting in hypoxia and cell death in deeper parts of the transplanted tissue. The imbalance in viable cell number at different parts of a thick tissue results in an inefficient cell integration and differentiation which leads to poor tissue functionality.^155^ This problem can be eliminated by either developing a well-organized vascular structure in engineered tissue where each cell can be within a diffusion limit or integrating host vasculature with grafted tissue through supermicrosurgery. With supermicrosurgery, it is possible to connect 300 to 800 *μ*m size vessels by using 30–80 *μ*m needles with microsutures. However, this procedure is time-consuming and requires extensive expertise and training.^156^ In one study, autologous fibroblast cells were seeded on a fibrous and porous hyaluronic acid scaffold. This skin patch was grafted into two different patients to show the efficacy for cutaneous wounds in two different case studies (1) skin removal for multiple epitheliomas, (2) chronic deep decubitus ulcer. In the first patient, after 1–3 weeks, the scaffold was fully integrated and showed vascularization as well. After 12 weeks of time, the patient's skin exhibited normo-elastic characteristics with no obvious scar formation. In the second patient, the ulcer was healed after eight weeks, and the implanted scaffold was fully resorbed, and complete re-epithelization was observed.^157^

Importantly, the use of stem cells plays a crucial role in the clinical translation of engineered tissues. MSCs were obtained from bone marrow of a patient and cultured in the blood serum of the patient. β-tricalcium phosphate (TCP) granules (1–3 mm diameter, 75% porosity, and 100–400 *μ*m pore size) were added to the cell suspension, and cells were cultured for two weeks further to induce osteogenic differentiation. The resulting vascularized fibula was implanted in a patient with bone necrosis. Post-surgery analysis showed re-vascularization at the treatment site.^158^ In another study, endothelial progenitors differentiated from type 1 diabetes mellitus patient iPSCs have been shown to assemble vascular network. The authors used engineered hyaluronic acid hydrogels to form 3D vascular networks *in vitro*. Then, they further showed the incorporation of these vascular networks to host vasculature in zebrafish as xenografts.^159^ In addition to stem cells, microvascular implants will also be important for this translation, especially when they are combined with tissue engineering applications. For instance, the vascular network of liver tissue was improved by co-culturing of HUVECs, MSCs, and iPSCs. Once its implanted into mice, successful integration into the host was achieved within days and the survival of mice was extended after liver injury.^160,161^

Despite several clinical studies reported, the number is still low due to the limitations of material certification and the availability of approved fabrication techniques. We believe that the future studies that focus on the development of innovative tissue engineering techniques, as well as interdisciplinary approaches, will generate continued motivation and hope for translatable strategies in clinics.

## IV. INTERDISCIPLINARY APPROACHES TO FACILITATE ANGIOGENESIS

Interdisciplinary research is on its way to facilitate angiogenesis. Extensive research is being carried out to synthesize custom-designed biomaterials to offer required biomechanical properties. On the other hand, fabrication techniques are being developed to provide optimum features in a scaffold, such as essential architecture and morphology to support vascularization.^162^ In this section, we will briefly review the advancements in biomaterials synthesis and scaffolding techniques and will critically evaluate the key challenges.

### A. Biomaterials

Natural matrices are constructed from polysaccharides [e.g., alginate, agarose, hyaluronic acid (HA), chitosan], and proteins (e.g., fibrin, collagen, gelatin, elastin). Hydrogels composed of these macromolecules adhere well to cells, and their cross-linking density and GF loading/release capacity can be altered by proteolytic degradation. When compared to synthetic scaffolds, natural matrices enable more significant interaction of cells with the host tissue, provide additional ECM components and increased cell deposition, sequester, and present GFs, and promote angiogenesis. The elemental composition of a natural material makes its processing easy and provides biocompatibility and has the potential to promote 3D microvasculature formation. However, it is generally difficult to modify these materials chemically and reproducibly, which restrict matrix characteristics such as biodegradability, rigidity, and the number of binding sites for GFs.

Moreover, there are ethical and clinical concerns over the use of animal-sourced material for therapeutic angiogenesis; since these components (e.g., decellularized animal tissues) can be highly immunogenic and can cause pathogen infection. Immunogenicity and the risk of infection from natural matrices have led tissue engineers to develop biodegradable synthetic biomatrices that recapitulate the natural ECM and its functions. Therefore, synthetic biomaterials have emerged as a more suitable alternative to natural matrices for translation to the clinic. Physical properties of synthetic biomaterials such as stiffness, elasticity, degree of cross-linking, along with the incorporation of cell adhesive ligands, GF-binding sites, and protease cleavage sites can be modified/controlled independently with high precision. These features provide reproducibility and enable the creation of porous 3D network designs specifically tailored to different biological applications. Synthetic matrices prepared with polymers such as poly(lactide-co-glycolide) (PLGA), polyethylene glycol (PEG), and peptide amphiphiles (PA) have thus become promising biological scaffolds for therapeutic angiogenesis.^163^ Different natural and synthetic biomaterials used in therapeutic angiogenesis studies are summarized in Table III,^125,154,164–184^ along with their composition, characteristics, and applications.

**TABLE III.** Natural and synthetic biomaterials being used to facilitate angiogenesis.

Natural biomatrices				
Biomaterial	Composition	Characteristics	Application and modifications	Example outcomes
Alginate	α-L-glucuronic acid and β-D-mannuronic acid	Non-toxic, temperature-independent	GF encapsulation in microspheres/beads and angiogenic induction, heparin conjugation	Slow and continuous FGF2 release and enhanced coronary circulation,^164^ sustained VEGF release from alginate beads and enhanced EC growth^165^
				Encapsulation of MSCs into Alginate-gelatin cross-linked hydrogel (ADA-GEL) microcapsules by means of AV loop^166^
Agarose	Agarose	Solid	GF release from beads and angiogenic induction, heparin conjugation	Local FGF2 release and augmented arteriogenesis^167^
				HUVECs sprouting withing Agarose (AG) + HA + Fibrinogen (FGN) microbeads promote vascularization^168^
Hyaluronic acid (HA)	N-acetyl-D-glucosamine and glucuronic acid	Anionic, non-sulfated, biodegradable, biocompatible	Controlled and sustained GF delivery from hydrogels and angiogenic induction, chitosan crosslinkage and adhesion peptides	Local and sustained VEGF and Ang-1 release from HA hydrogels, enhanced vessel sprouting and maturation^169^
Chitosan	D-glucosamine and N-acetyl-D-glucosamine	Biocompatible, biodegradable, water-soluble, bioadhesive	GF encapsulation in microspheres and angiogenic induction, HA, or collagen crosslinkage	Sustained and controlled FGF1 release and promoted neoangiogenesis^170^
				Platelet-rich plasma (PRP)-Chitosan hybrid induce angiogenesis^171^
Fibrin	Fibrin monomers	Proangiogenic, weak, injectable	GF encapsulation or covalent conjugation in fibrin gel and angiogenic induction, heparin conjugation	Controlled and sustained covalently conjugated VEGF delivery, augmented local neovascularization^172^
				T1-functionalized fibrin hydrogels promotes therapeutic vascularization^173^
Collagen and gelatin	Collagen type I	Lower loading capacity, strong, biocompatible, biodegradable, permeable, porous, proangiogenic	GF encapsulation or covalent conjugation and angiogenic induction, heparin crosslinkage, chitosan crosslinkage and adhesion peptides	Prolonged and local VEGF delivery,^174^ augmented FGF2-mediated HUVEC growth and vascularization^175^
				BMP-2 delivery with decorin-supplemented collagen hydrogels for revascularization of bone-muscle injury^176^
Elastin	Elastin monomers and microfibrils like fibrillin	Flexible	Angiogenic induction, heparin crosslinkage	Enhanced vessel sprouting by activated ECs and pericytes^177^
Matrigel	Various ECM molecules such as collagen type IV, entactin and laminin	Proangiogenic, biocompatible, biodegradable, solid	*In vitro* EC culturing and capillary tube formation, *in vivo* angiogenesis and 2D and 3D tissue network mimicking	Augmented 2D solid endothelial cord networks with MSCs in rat aorta culture^178^
Microspheres	Natural polymers such as alginate, chitosan, and gelatin	Biocompatible, biodegradable, high surface-to-volume ratio, micro and nano-scale production, hydrophilic	Angiogenic induction, local and sustained delivery of encapsulated GFs, heparin crosslinkage	Local and prolonged VEGF release from calcium alginate microspheres and augmented microvessel formation^125^
				VEGF co-culture with dental pulp stem cells (DPSCs) to promote blood vessels and pulp-like tissue^179^
Synthetic biomatrices				
Biomaterial	Composition	Characteristics	Application	Outcomes
PLGA	Lactide and glycolide polyesters	Bioadhesive, water-insoluble, solid, porous, amorphous, biodegradable	Angiogenic induction by encapsulating or conjugating GFs, cells and ECM molecules, controlled single or multiple GF release, heparin crosslinkage and adhesion peptides	Distinct release rates of VEGF and PDGF-BB, their sustained and local release and augmented vascularization and maturation^154^
				PLGA-based bioartificial devices pre-vascularized with hypo-MSCs^180^
PEG hydrogel	PEG macromers	High viscoelasticity, biodegradable, biocompatible, water-soluble, hydrophilic, non-adhesive, photopolymerisable	ECM mimicking, angiogenic induction, avoidance of non-specific cell adhesion and intimal thickening, controlled release of encapsulated or chemically conjugated GFs, heparin crosslinkage, adhesion peptides, proteolytic cleavage sites	Augmented EC and SMC proliferation, HUVEC migration and angiogenesis by controlled and local delivery of covalently immobilized PDGF-BB^181^
				HUVECs and 10T1/2 co-culture within PEG hydrogel promotes prevascularization^182^
Peptide amphiphiles (PA)	Amino acids sequence and alkyl tail	Biocompatible, biodegradable, soft, hydrophobic, nanofiber formation, pH-sensitive	ECM or GF activity mimicking, angiogenic induction, heparin crosslinkage and adhesion peptides, hierarchical organization	Augmented neovascularization by controlled VEGF and FGF2 release from heparin-crosslinked gels^183^
Microspheres	Synthetic polymers such as PLGA and polycaprolactones	Biocompatible, biodegradable, high surface-to-volume ratio, micro- and nano-scale production, lipophilic	Angiogenic induction, local and sustained delivery of encapsulated GFs, heparin crosslinkage	Enhanced blood vessel formation and angiogenic chemokine expression by controlled release of PDGF-BB from PLGA microspheres^184^

### B. Preparation techniques

A biomaterial, either natural or synthetic, merely provides a favorable surface phase for biological system interaction, but how this material's surface looks like depends on in what way it has been processed. Further, when we talk about the need for an organized, patterned, or well-established network, the fabrication technique being utilized becomes crucial. Over time, existing techniques are getting modernized, and some new procedures and methods are being introduced to cope with the challenge of vascularization. Here, we will discuss some of the leading scaffold fabrication techniques in detail. The selection of a particular technique to process a biomaterial solely depends on the intended use of developed scaffold or tissue. Sometimes, a combination of more than one may serve the purpose as well. For example, Kazimierczak and co-workers^185^ combined relatively simple and cost-effective methods together (freeze-drying and gas foaming) to develop a fairly optimum scaffold possessing a high share of macropores. These interconnected macropores are beneficial for vascularization and bone ingrowth *in vivo*.^186^ In another recent approach,^187^ polycaprolactone and collagen were processed through 3D printing and on top of these struts providing topographical cues, nanofibers of HUVECs-laden alginate bioink were deposited through electrospinning. Myoblast cells were seeded on these composite structures, and scaffolds containing coculture of HUVECs and myoblast showed myoblast regeneration.

#### 1. Electrospinning

Electrospinning is an efficient method to develop scaffolds with nanometer-sized fibers. These scaffolds have ECM-like architecture, adjustable porosity, high surface/volume ratio, tunable surface properties, and may have a 3D interconnected network as well.^188^ The ease of process allows to incorporate desired moieties for inducing vascularization, biomaterial modification, and well control of physical and mechanical properties of the scaffold through process parameters as well.^189^ Although many successful accounts have been reported in the literature regarding enhanced vascularization in electrospun scaffolds,^51,190–193^ this technique can generally produce scaffolds with a limited thickness (usually tens to hundreds of a micrometer), and typically it is challenging to control precise dimensions and morphology of a 3D scaffold.^194,195^ However, thickness issues can be overwhelmed by combining the electrospun membrane with other scaffolds such as hydrogel.^196^

#### 2. Spatial patterning and lithography

Lithography is a microfabrication technique that enables the manufacture of precise and complex 2D or 3D structures with tiny features (below 10 nm).^197^ In a typical procedure, a photo-sensitive material known as photoresist (PR) is spin-coated on a silicon wafer or substrate. A pattern present on a photomask is transferred onto the substrate through exposure. Exposed areas on photoresist are then retained or removed depending on the composition of the photoresist (i.e., positive or negative PR).^198^ Microscale devices developed through lithography have also been used and analyzed for their vascularization potential.

**FIG. 9.**
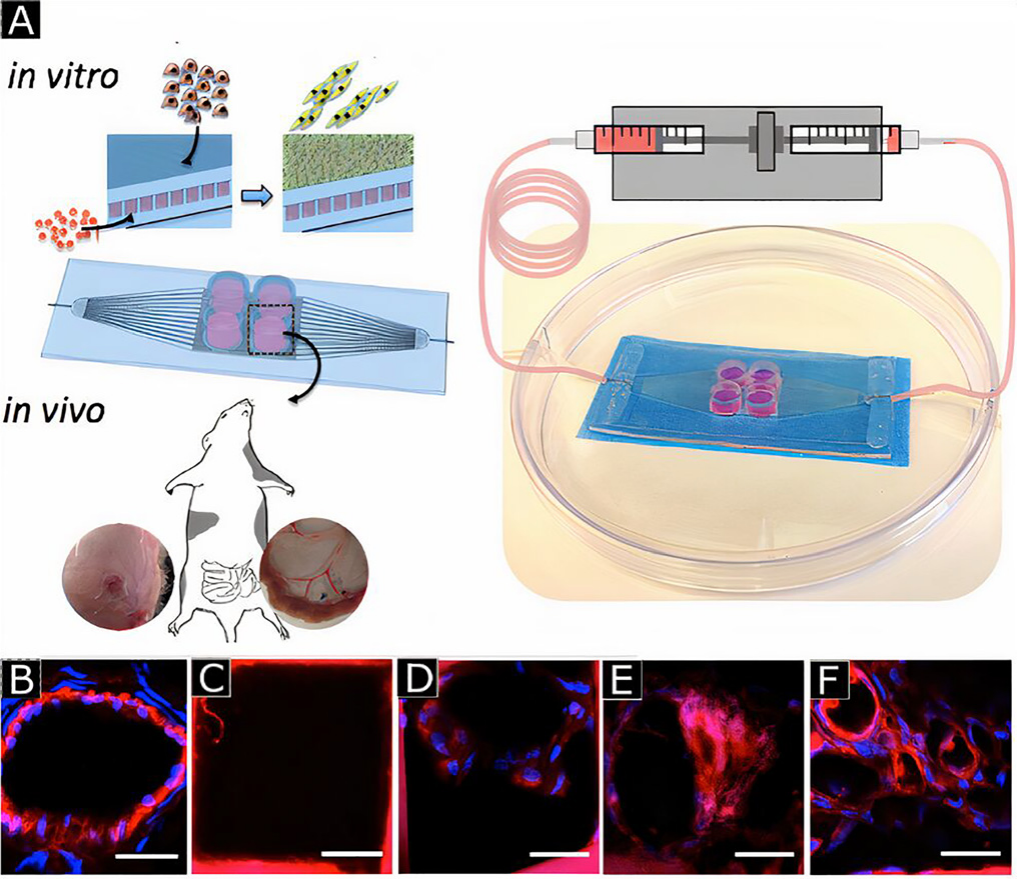
Experimental design for *in vitro* and *in vivo* studies (a), where human skeletal muscle-derived cells and human umbilical vein endothelial cells were seeded in parenchymal spaces and in microvessels respectively while for *in vivo* studies, full-thickness punches of scaffolds with/without cells were implanted in nude rats. Immunostaining with anti-rat CD31 for a native blood vessel (b), Subcutaneous (SC) scaffold without (c) or with cells (d), and IP scaffold without (e) and with cells (f). Scale bar: 25 *μ*m. Reprinted with permission from Ye *et al.*, Biomaterials **34**, 10007 (2013). Copyright 2013 Elsevier.

Ye *et al.* devised a microvessel scaffold from biodegradable poly (glycerol sebacate) using soft lithography. The scaffold device was designed in the shape of a boat with 250 *μ*m-diameter inner and outer channels. Boat's central area was 4 cm^2^ and had 150 parallel channels. Channels in the central region were set as 100 × 100 *μ*m^2^ to mimic small blood vessels. Cells were seeded onto these microvessels *in vitro* [Fig. 9(a)], and full-thickness scaffold punches were implanted in nude rats. After one week of implantation, vascularization was observed in the microchannels [Figs. 9(b)–9(f)]. Further, *in vivo* implantation of the device in nude rats resulted in biodegradation through surface erosion and infiltration of host blood cells into the microvessels.^199^ Similarly, Narbat *et al.* developed a method for patterning cells by lithography.^200^ They used HUVECs and MSCs in methacrylated-gelatin (GelMA) solution containing VEGF. They exposed the system to light by using a patterned photomask and performed partial photo-polymerization at the exposed areas only. Subsequently, preosteoblast cells containing GelMA was patterned on top of HUVECs and MSCs laden GelMA stripes. This way, a spatially patterned cell surface resembling to a vascular tree was achieved. This approach helped to control vascular structure with a pattern size of <150 *μ*m in width. Differentiated mural cells stabilized vascular structures, while osteoblasts maintained osteogenic potential of the tissue. By using this method, it is possible to develop tissue with multiple cell niches. An AngioChip with interconnected lumens was developed by Zhang *et al.* by using poly(octamethylene maleate (anhydride) citrate) POMaC. POMaC was used to provide better elasticity and ease of processing. 3D branched network was developed in a layer-by-layer fashion. These micro/nanopores enhanced permeability and intercellular interactions. Vascularized cardiac tissues were engineered through hESC-HUVECs and hESC-derived cardiomyocytes with 10% hMSCs as supporting cells by using AngioChip.^201^

#### 3. Self-assembly of microvascular network

**FIG. 10.**
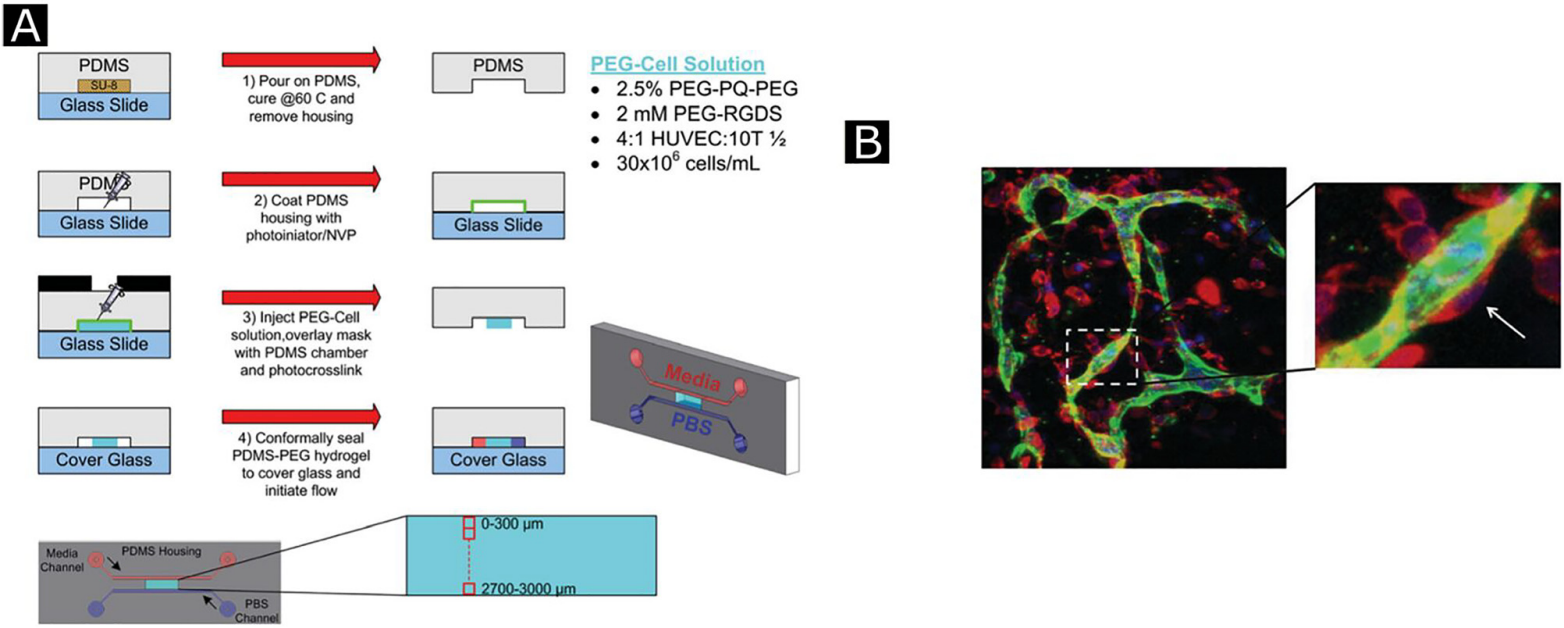
Schematics of microfabrication design (a), in step 1, to fabricate an external perfusion housing, PDMS is replica molded. In step 2, the interior of the housing is coated with a photoinitiator. In step 3, the housing is injected with photopolymerizable PEG precursors, and to fabricate hydrogel microchannels within the external PDMS housing, mask*-*directed photolithography is used. In step 4, the PDMS–PEG multilayer device is conformally sealed to coverglass and perfused with media and buffer. The last schematic shows the spatial relationship of the perfused media (red) and buffer (blue) microchannels to PEG hydrogel (cyan) regions imaged for analysis. Immunohistochemistry (IHC) of microvascular network formation at 96 h (B), where HUVECs (green), 10T1/2 cells (red), and nuclei (blue) Reprinted with permission from Cuchiara *et al.*, Adv. Funct. Mater. **22**, 4511 (2012). Copyright 2012 John Wiley & Sons, Inc.

The idea of self-assembly is a generation of a structure in which the newly formed state is thermodynamically stable because of the non-covalent interactions such as hydrogen bonding, ionic, electrostatic, hydrophobic, and van der Waals interactions. These nanofibers, with a diameter of 1000 nm or below, can be obtained with amino acids.^202^ In addition to amino acids, natural or synthetic polymers can also be used for fabrication of self-assembled nanofibers. For instance, Cuchiara and co-workers used PEG-based hydrogels to form prevascularized perfusable tissue.^182^ Authors prepared microfluidic PEG hydrogel within a polydimethylsiloxane (PDMS) housing by using soft lithography and photolithography techniques [Fig. 10(a)]. The tubule networks could be observed through the use of HUVEC and mesenchymal progenitor (10T1/2) cells *in vitro* [Fig. 10(b)].

**FIG. 11.**
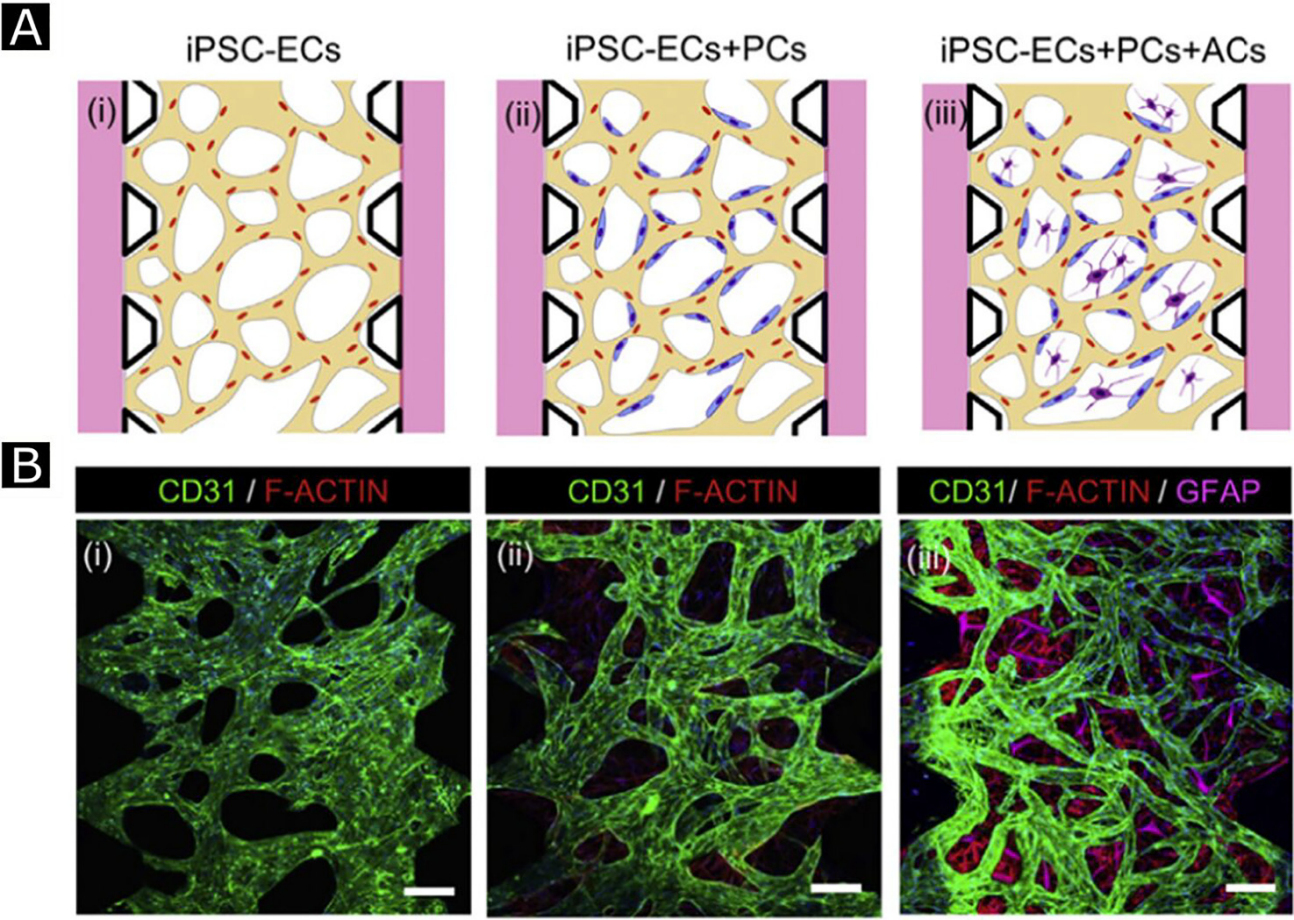
Schematics of microvascular network conditions for optimization of self-assembly of microvasculature (a), where (i) iPSC-ECs only, (ii) iPSC-ECs + PCs, (iii) iPSC-ECs + PCs + ACs. Confocal images of microvascular networks after 7 days (b), where (i) iPSC-ECs only (CD31, green), (ii) co-culture with PCs (F-actin, red), (iii) tri-culture with ACs [Glial fibrillary acidic protein (GFAP), magenta]. Scale bars indicate 100 *μ*m. Reproduced with permission Campisi *et al.*, Biomaterials **180**, 117 (2018). Copyright 2018 Authors, licensed under a Creative Commons Attribution (CC BY) license.

Another approach was developed to demonstrate self-organization of microvascularized blood–brain-barrier model by using ECs, PCs, and astrocyctes (ACs).^203^ The authors demonstrated that vessel formation with induced pluripotent stem cell (iPSC)-ECs only was not possible after 7 days whereas co-culturing of iPSC-ECs with PCs led to small and highly branched vessels that are similar to the morphology in natural microenvironment. This model was then further improved by introducing ACs to create a complex inter-connected and branched vessel network that would resemble in the natural microenvironment *in vivo* (Fig. 11).

#### 4. 3D bioprinting

**FIG. 12.**
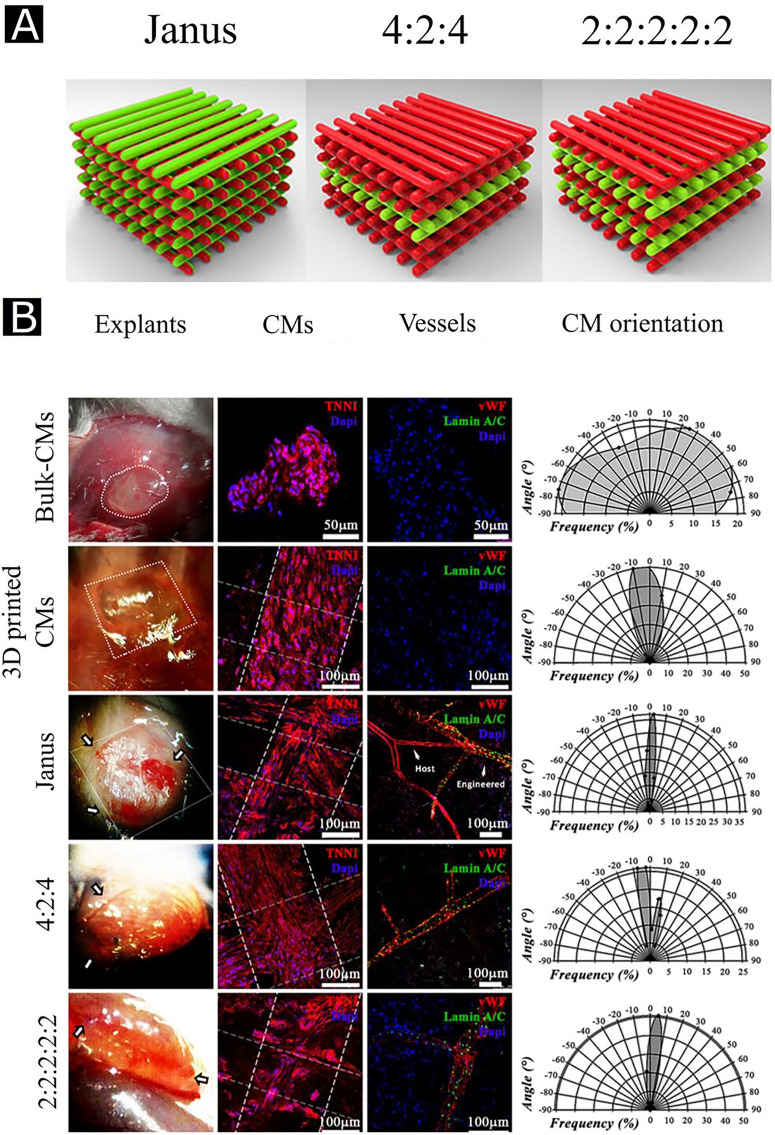
Explants analyses after two weeks of implantation. Schematics for multi cells containing 3D bioprinted cardiac tissue constructs with three different geometries. (a) *In vivo* grafting of bulk and 3D bioprinted hydrogels after 15 days of implantation (first column from left), immunofluorescence staining to visualize CMs orientation (second column), and vessels (third column) while polar graphs are quantifying CMs orientation (fourth column). Arrows indicate vascularization, dash lines in column explant shows implantation site, dash lines in column CMs shows the organization of vasculature. TNN1, DAPI (4′,6-Diamidino-2-phenylindole dihydrochloride), vWF, and Lamin A/C were used to stain CM, nucleus, vasculature, and capillaries originated from human endothelial cells, respectively (b). Reproduced with permission from Maiullari *et al.*, Sci. Rep. **8**, 13532 (2018). Copyright 2018 Authors, licensed under a Creative Commons Attribution (CC BY) license.

The advancement in fabrication technologies has enabled the application of three-dimensional (3D) printing in manufacturing, engineering, and medicine. 3D bioprinting is gaining importance than conventional fabrication techniques for tissue engineering and regenerative medicine applications. This technique facilitates to deposit bioink consisting of cells, biomaterial, or combination of both in layer-by-layer fashion to build a 3D structure with required architecture and characteristics. This structure can be pre-designed through computer-aided design (CAD) software according to defect size and dimensions.^204^ The conventional methods to produce porous structures include gas foaming, porogen leaching, or freeze-drying. However, pore size and interconnectivity of pores cannot be controlled precisely in these methods, which is an essential requirement for vascularization, whereas 3D bioprinting provides this opportunity to achieve a well-established architecture.^205^ Commonly available 3D printing-based technologies have shown potential in developing vascularized models.^206,207^ Further, minimum vessel sizes of ∼200 *μ*m, ∼100 *μ*m, and ∼1 *μ*m have been reported through inkjet-, extrusion-, and light-based modalities.^208^ Therefore, we can print blood vessels ranging from arteries to veins with the help of current technology, but the associated cost can go much higher, especially with light-assisted technology. Still, high hopes associated with 3D bioprinting have driven researchers to explore this opportunity to solve the ever-existing angiogenesis issue with 3D printed scaffolds. Kim *et al.*^209^ used ECM derived from the skin (dECM) as bioink for 3D printing of full-thickness (1 mm) skin graft consisting of native cytokines and GFs. Apart from cytokines and GFs, dECM has an added advantage over collagen printed graft, the later shrinks in *in vitro* conditions. EPCs and adipose-derived stem cells (ASCs) laden 3D printed skin patch grafted in mice showed re-epithelization and neovascularization, which resulted in blood flow measured by Doppler perfusion imaging. EPCs and ASCs laden scaffold exhibited better blood flow than control and acellular dECM scaffold. These results show that spatially controlled and laden cells in 3D printed scaffolds can induce neovascularization and can prevent the possibility of hypoxia in full-thickness grafts. Maiullari and co-workers^210^ recently developed a 3D cardiac patch through 3D printing of HUVECs and iPSCs derived cardiomyocytes (CMs) encapsulated in alginate and PEG-fibrinogen. Patches were printed having three different geometrical architectures: (1) both cells were uniformly distributed within each deposited fiber layer (termed as Janus), (2) alternating two layers of HUVECs with two layers of CMs, and (3) alternating two layers of HUVECs with four layers of CMs [Fig. 12(a)].

Developed tissue was implanted in mice, and after two weeks, explant was analyzed. Results demonstrate that the blood vessels generated in the tissues where multi cells were present and vessels were well-integrated with the host vasculature as compared to alone CMs. Branched vascular capillaries were formed with well orientation and organization of CMs were evidenced by polar graphs [Fig. 12(b)]. 3D printing technology, being a versatile fabrication technique, also allows the processing of inorganic materials. For example, a study^211^ shows enhanced blood vessel formation during *in vivo* bone regeneration in 3D printed porous scaffolds comprising of iron oxide and silica doped tricalcium phosphate (TCP) particles without the use of any GF. 3D printed scaffolds exhibited good compressive strength (∼20 MPa in doped scaffolds) and a pore size of around 300 *μ*m after sintering. After eight and 12 weeks of implantation, the analysis was performed. Iron and silicon doped TCP scaffolds showed collagen production, bone regeneration, and enhanced neovascularization at host bone interface. Conclusively, we can say, that 3D bioprinting technique enables to fabricate scaffolds with much-controlled architecture, design, porosity, and ease of using a range of biomaterials.

## V. CHALLENGES AND OPPORTUNITIES IN NEOVASCULARIZATION

Despite the significant progress made in the field, the generation of thick tissues with functional microvasculature *in vitro* still poses a significant challenge that needs to be addressed before it can become a viable alternative in clinical tissue/organ transplantation. According to Zhang and Radisic,^212^ organ-level vascularization is just like a Mars mission in bioengineering, which requires much preparation. One of the remaining challenges in angiogenic therapy is the risk of undesired and uncontrolled tissue ingrowth. Uncontrolled delivery of many GFs such as FGF-1, FGF-2, and PDGF-BB can trigger neointimal thickening by excessively enhancing the proliferation and migration of SMCs. The resulting intimal thickening can lead to stenosis or restenosis, which is the abnormal restriction of blood vessels and, eventually, circulation and regress the tissue into the ischemic state. GF dosage should also be carefully determined. Delivery of single or multiple GFs in large doses may disrupt vessel stability. For instance, excess FGF-1 may rupture plaques within vessels and lead to atherosclerosis with vessel instability and breakage.^213^ To eliminate these problems, future studies should prioritize optimizing multiple GF delivery so that these undesired side effects can be reduced or eliminated. For each GF, an optimal concentration range should be determined where the GF concentration is sufficiently high to exert biological activity, yet lower than concentrations that cause the mentioned undesired results such as tumorigenesis.

In GF delivery using biomimetic material scaffolds, improvements are needed to control GF release from functionalized biomaterials to recapitulate the natural microenvironment. Fabrication techniques should be in line to control required physical properties (e.g., pore size, water content, porosity, the interconnection of pores, etc.) of engineered scaffold for proper vascularization. In reported studies, cells are placed within matrices, and they are free to generate vascular structures on their own fate that usually results in unwanted architecture and patterns. Advanced techniques like electrospinning, patterning, and 3D printing can be combined for providing scaffold-guided cell path to mimic natural architecture of a tissue which is necessary for vascularization.^214,215^ Translation to the clinic remains another major challenge as such applications are also hampered by the lack of experience in GF administration of clinicians in phase I/II clinical trials.^50^ As the effectiveness of bioactive factors in *in vitro* and *in vivo* conditions are different, therefore, optimal properties of GF delivery from scaffolds cannot be decided based on a few successful clinical studies. Future clinical studies should focus on the integration of pre-vascularized engineered tissue with host vasculature through advanced supermicrosurgery, and additionally, implanted tissue can release multiple GFs dynamically with spatiotemporal control.^50^

In therapeutic angiogenesis, another growing area is to find new target proteins and genes within signaling pathways that regulate angiogenesis. Such components of angiogenic pathways can be identified and characterized well by using new proteomic and genomic methods.^71^ For example, tumor angiogenesis can be repressed, or neovascularization can be induced using miRNA targeting, and nucleic acids that enhance the activity and proliferation of proangiogenic GFs may be overexpressed.

In addition to the challenges mentioned above, the following issues determine the success of vasculogenesis; (1) finding appropriate cells from a donor tissue along with the number of cells needed during implantation to promote vasculogenesis, (2) interaction of implanted cells with the host immune system, (3) integration of implanted cells with the host tissue microenvironment. Since, the isolation of terminally differentiated ECs from patients requires invasive techniques, finding proper cell source is still a challenge. In addition, ECs are not sufficient to recapitulate the natural microenvironment of a vascularized tissue. Therefore, different cell types are required to mimic the heterocellular microenvironment of the native tissue *in vivo*. To address this issue, recently, iPSCs have been considered due to their unique differentiation capability to promote vascularization.^215^ The initial number of cells implanted is critical to ensure the functionality of the engineered tissue after transplantation.^216,217^ To overcome an undesired immune response, either the “immonuevasive” microvascular tissue engineering approach or the host's own cells can be used.^216^ In the “immunoevasive” approach, ECs can be genetically engineered by using CRISPR/Cas9 to ablate major histocompatibility complex (MHC) class II molecule from the surface of the ECs. Transplanted tissue that is generated with CRISPR-modified ECs circumvents the T-cell recognition. Therefore, acute rejection of transplanted tissue would be hindered.^217^ HUVECs have been commonly used to promote vascularization both *in vitro* and *in vivo*. However, the new vasculature that forms upon HUVEC implantation is limited to integration with the existing vasculature at the implantation site. The use of EPCs and organ-specific ECs have been considered to overcome the difficulty associated with the integration of new vasculature at the implantation site.^12^

Although many recent studies were focused on these problems, definitive solutions are yet to emerge. Successful clinical translation of engineered grafts would only be possible through combining tissue engineering techniques (cells, decellularized tissue, and GF delivery) and inter-disciplinary systems (functionalized biomaterials and fabrication techniques). We believe these issues can be addressed to promote vascularization of transplanted tissues, so that clinical translation of engineered grafts would be possible.

## AUTHORS' CONTRIBUTIONS

M.A.N. and I.C.K. contributed equally to this work.

## Data Availability

The data that support the findings of this study are available from the corresponding author upon reasonable request.

## NOMENCLATURE

AC: Astrocyte
Ang-1: Angiopoietin 1
Ang-2: Angiopoietin 2
ASC: Adipose-derived stem cell
AV: Arteriovenous
CAD: Computer-aided design
CAM: Chorioallantoic membrane
CM: Cardiomyocytes
EC: Endothelial cell
ECM: Extracellular matrix
EDC: 1-ethyl-3–(3-dimethylaminopropyl)carbodiimide
EPC: Endothelial progenitor cell
FGF-2: Fibroblast growth factor 2
GAG: Glycosaminoglycans
GelMA: Methacrylated-gelatin
GF: Growth factor
hESC: Human endothelial stem cell
HIF-1α: Hypoxia-inducible factor 1α
hMSC: Human mesenchymal stem cell
HSC: Hematopoietic stem cell
HUVEC: Human umbilical vein endothelial cells
IA: Intra-arterial
IGF: Insulin-like growth factor
iPSC: Induced pluripotent stem cell
IV: Intravenous
MMP: Matrix metalloproteinases
NHS: N-hydroxysuccinimide
NO: Nitric oxide
NOS: Nitric oxide synthase
PA: Peptide amphiphiles
PC: Pericyte
PDGF: Platelet-derived growth factor
PDMS: Polydimethylsiloxane
PEG: Polyethylene glycol
PEGDA: Polyethylene glycol diacrylate
pI: Isoelectric point
PLGA: Poly(lactide-co-glycolide)
pNIPAAM: Poly(N-iso-propylacrylamide)
POMaC: Poly(octamethylene maleate (anhydride) citrate)
PR: Photoresist
SDF-1: Stromal cell-derived factor 1
SMC: Smooth muscle cell
TCP: Tricalcium phosphate
TGF: Transforming growth factor
UV: Ultraviolet
VEGF: Vascular endothelial growth factor
VSMC: Vascular smooth muscle cell
3D: Three-dimensional
